# Effect of curcumin analogs onα-synuclein aggregation and cytotoxicity

**DOI:** 10.1038/srep28511

**Published:** 2016-06-24

**Authors:** Narendra Nath Jha, Dhiman Ghosh, Subhadeep Das, Arunagiri Anoop, Reeba S. Jacob, Pradeep K. Singh, Narasimham Ayyagari, Irishi N. N. Namboothiri, Samir K. Maji

**Affiliations:** 1 Department of Biosciences and Bioengineering, Indian Institute of Technology Bombay, Mumbai 400 076, India; 2 IITB Monash Research Academy, Indian Institute of Technology Bombay, Mumbai 400 076, India; 3 Department of Chemistry, Indian Institute of Technology Bombay, Mumbai 400 076, India

## Abstract

Alpha-synuclein (α-Syn) aggregation into oligomers and fibrils is associated with dopaminergic neuron loss occurring in Parkinson’s disease (PD) pathogenesis. Compounds that modulate α-Syn aggregation and interact with preformed fibrils/oligomers and convert them to less toxic species could have promising applications in the drug development efforts against PD. Curcumin is one of the Asian food ingredient which showed promising role as therapeutic agent against many neurological disorders including PD. However, the instability and low solubility makes it less attractive for the drug development. In this work, we selected various curcumin analogs and studied their toxicity, stability and efficacy to interact with different α-Syn species and modulation of their toxicity. We found a subset of curcumin analogs with higher stability and showed that curcumin and its various analogs interact with preformed fibrils and oligomers and accelerate α-Syn aggregation to produce morphologically different amyloid fibrils *in vitro*. Furthermore, these curcumin analogs showed differential binding with the preformed α-Syn aggregates. The present data suggest the potential role of curcumin analogs in modulating α-Syn aggregation.

α-synuclein (α-Syn) is a 140 amino acid residue protein (~14 kDa) expressed at high levels in neurons^1^,^2^. Fibrillar aggregates of α-Syn inside the dopaminergic neuron is the major component of Lewy bodies and Lewy neurites inclusion, which are considered as pathological hallmark of Parkinson’s disease (PD)^3^. Direct involvement of α-Syn aggregation in PD pathogenesis have been suggested in several *in vitro* and *in vivo* studies^3^,^4^. α-Syn is a natively unfolded protein shown to aggregate and form amyloid fibrils *in vitro*^5^,^6^,^7^,^8^,^9^.

The α-Syn, during amyloid aggregation undergoes structural assembly from monomeric to fibrillar states through oligomeric intermediates. Both *in vitro* as well as *in vivo* studies suggest that the soluble, oligomeric forms of α-Syn are potent neurotoxic species responsible for the neuronal injury and death of neurons in PD^10^,^11^,^12^. Therefore, molecules that inhibit the toxicity of oligomers either by reducing their formation or by converting them to less-toxic or non-toxic state would be an effective agent for the drug development against PD^13^,^14^,^15^. Based on this idea, several researchers have searched for chemically synthesized or naturally existing small molecules acting as inhibitors of α-Syn fibrilogenesis^14^,^16^,^17^,^18^,^19^,^20^,^21^.

Curcumin (diferuloylmethane) is a well-known naturally occurring polyphenolic constituent present in turmeric and has demonstrated anti-carcinogenic, anti-microbial and anti-inflammatory activities^22^,^23^. As curcumin is a small molecule with ability to cross the blood brain barrier and therapeutically active, it is now considered one of the most promising candidates in the treatment of various neurological disorders^24^,^25^,^26^. Furthermore, recently it has been shown that curcumin binds to preformed fibrils/oligomers modifying their exposed hydrophobic surface, hence reducing their toxicity^27^. Therefore, curcumin and other related polyphenolic compounds could be used as drug for the treatment of PD and other neurological diseases. In the present work, we selected various curcumin analogs, which could possess higher potential to modulate the α-Syn aggregation and/or decrease their cytotoxicity. Our current study show that curcumin analogs with high efficiency to modulate α-Syn aggregation may have potential therapeutic value against PD.

## Results

### Selection of curcumin analogs

**Figure 1:**
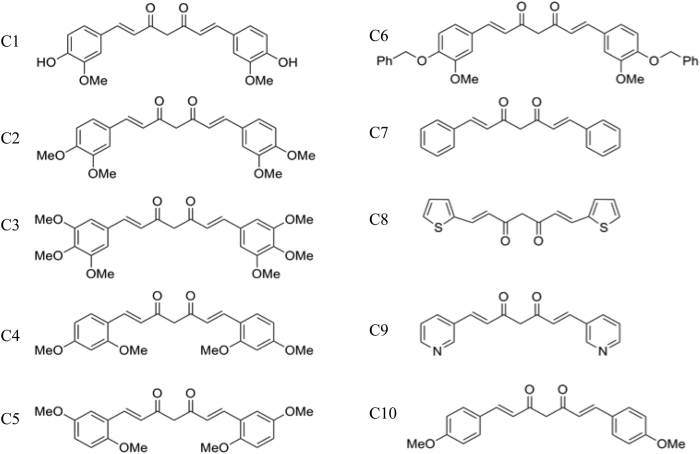
Chemical structures of curcumin (1) and its analogs (2–10) used in this study.

It has been shown that two aromatic groups in curcumin separated by a planar backbone are important to fulfill its role on modulating the aggregation process and amyloid formation^28^,^29^,^30^. Therefore, we hypothesized that chemical modification of this aromatic group would be helpful to further increase its efficiency. Previous study from our group showed reduction in cellular toxicity of α-Syn aggregates owing to minimized hydrophobic surface exposure due to binding of curcumin^27^. In our current study, we preferentially selected (from already synthesized curcumin analogs library^31^,^32^,^33^) some curcumin analogs with different substitution on the aromatic ring that could affect the hydrophobicity of parental compound and thereby will help to bind with the oligomers and fibrils with a greater extent. In order to alter the hydrophobicity of the analogs, the compounds were selected in such a way that hydroxyl group of curcumin was replaced by −OMe (C2, C4 and C5) and −OCH_2_Ph (C6) groups (Fig. 1). Furthermore, we also choose a compound with one additional −OMe group in each aromatic ring to make it more hydrophobic (C3). As a control, we used curcumin without −OH group (C10) and other substituent (C7) attached to aromatic rings. To further examine the role of aromatic ring in curcumin, we selected compounds with two hetero-nuclear aromatic moieties (C8 and C9). As it was shown that the free hydroxyl groups of curcumin attached to the aromatic ring are modified by liver, kidney, and intestinal mucosa, produces curcumin glucuronide and curcumin sulfate, which results in low bioactivity^34^,^35^,^36^, we expect that the selected analogs (−OH substituted with −OMe and −OCH_2_Ph) might show higher bioactivity. All these compounds were synthesized by condensation of acetyl acetone with aromatic aldehyde and the products were purified by re-crystallization or by silica gel column chromatography as reported earlier^31^,^37^,^38^,^39^,^40^,^41^.

### Spectroscopic properties of Curcumin analogs

The spectral property of curcumin has been well studied previously^27^. The absorption maximum of curcumin is ~425 nm^27^. When excited at 425 nm, curcumin gives maximum fluorescence emission at ~535 nm^27^. To characterize the spectral properties of curcumin analogs, all the analogs were dissolved in dimethylsulfoxide (DMSO) and then diluted in 20 mM MES (2-(N-morpholino)ethanesulfonic acid) buffer, pH 6.0. For determining absorption property, the UV absorption spectra of all the curcumin analogs (40 μM) were recorded in the range of 300–600 nm. The analogs showed absorption maxima within the wavelength range of 382 nm to 438 nm (Figs S1A and S1B). For fluorescence spectroscopic properties, 50 μM of all the curcumin analogs were prepared in 20 mM MES buffer, pH 6.0. Then each curcumin analog was excited at their maximum absorption wavelength and the emission spectra were recorded. When excited at 425 nm, curcumin gives fluorescence maximum at 533 nm. However, each curcumin analog showed different fluorescence spectra with difference in their fluorescence emission intensity (Figs S1A and S1B).

### Stability and toxicity of curcumin analogs

**Figure 2:**
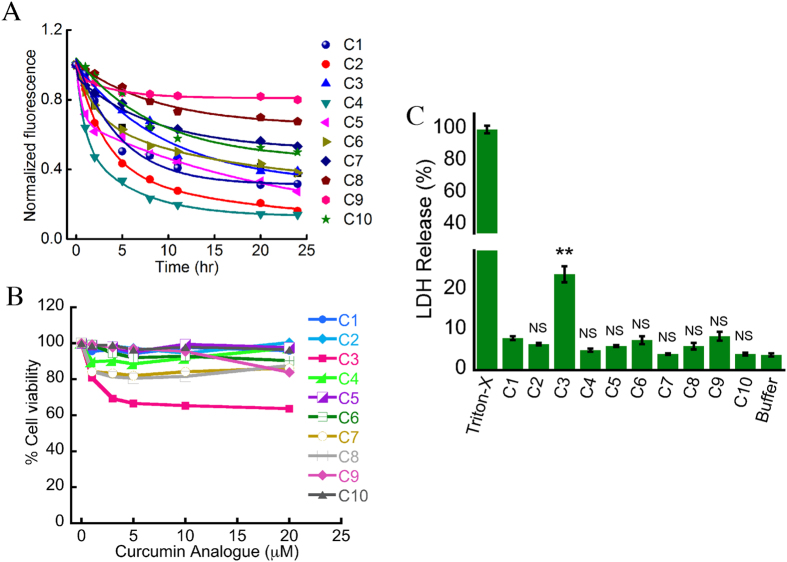
Stability and toxicity of curcumin analogs. (**A**) Plot of normalized fluorescence intensity versus time. The slope of C2 and C4 are steeper than curcumin, suggest that C2 and C4 are less stable than curcumin while other analogs with reduced slopes are more stable than curcumin under similar conditions. (**B**) MTT assay of different concentrations of analogs ranging from 0–20 μM. The analog number 3 (C3) showed the most toxic effect, while others showed similar extent of toxicity as compared to curcumin (C1). (**C**) LDH cytotoxicity assay of curcumin and its analogs at 20 μM. All the analogs, except C3 showed less than 10% toxicity. The bar diagram of LDH cytotoxicity assay represents mean ± SE from three independent set of experiments (for each experiment triplicate wells were used).

Curcumin is less stable in buffer near neutral pH (PBS 7.4), however at slightly acidic pH, curcumin possesses better stability^42^,^43^. Therefore, we used pH 6.0 (MES buffer) for our study. The stability of synthetic curcumin analogs were studied in comparison with curcumin by time-dependent changes in intrinsic fluorescence in 20 mM MES buffer (pH 6.0, 0.01% NaN_3_). The normalized fluorescence value for each curcumin analog versus time was plotted and the slope of the plots provides the relative stability of the curcumin and its analogs. The decrease in fluorescence intensity with time reveals that the slope of C2 and C4 are steeper as compared to curcumin suggesting that C2 and C4 are less stable than curcumin (Figs 2A and S2). The other analogs (C3, C5, C6, C7, C8, C9 and C10) showed shallower slope and therefore are relatively more stable than curcumin under similar conditions (Figs 2A and S2). Interestingly, C5 does not follow smooth exponential decay compared to others. It could be because the initial decrease in fluorescence intensity for C5 was much faster as compared to the later time points. This suggests that at the initial time points, C5 degrades much faster than its corresponding latter time points. Also it is important to note that even after 24 h of incubation, fluorescence intensity of C5 continues to decrease while all the others almost reach to saturation.The data collectively suggest most of the curcumin analogs used in the present study are more stable as compared to curcumin.

To study the potential cytotoxicity of curcumin analogs, all compounds were tested for MTT (3-(4,5-dimethylthiazol-2-yl)-2,5-diphenyltetrazolium bromide) reduction assay^44^ and lactate dehydrogenase (LDH) release assay^45^ using SH-SY5Y cells. The analogs were tested with different concentrations ranging from 0–20 μM for the MTT assay. All analogs except C3 showed more than 80% cell viability (<20% cytotoxicity) in MTT assay, indicating they are less cytotoxic in nature (Fig. 2B); analog C3 showed ~40% toxicity. To further validate the results of MTT assay, we performed the LDH cytotoxicity^45^ assay of curcumin and its analogs. Based on MTT assay, we have used highest concentration (20 μM) of curcumin and its analogs for LDH assay. Similar result was also observed in LDH release assay (Fig. 2C), where C3 showed maximum cell death (~25%) and the remaining analogs showed less than 10% cell death (Fig. 2C). The data suggest that all the curcumin analogs (except C3) are non-toxic under the conditions studied.

### Effects of curcumin analogs on preformed α-synuclein amyloid fibrils

**Figure 3:**
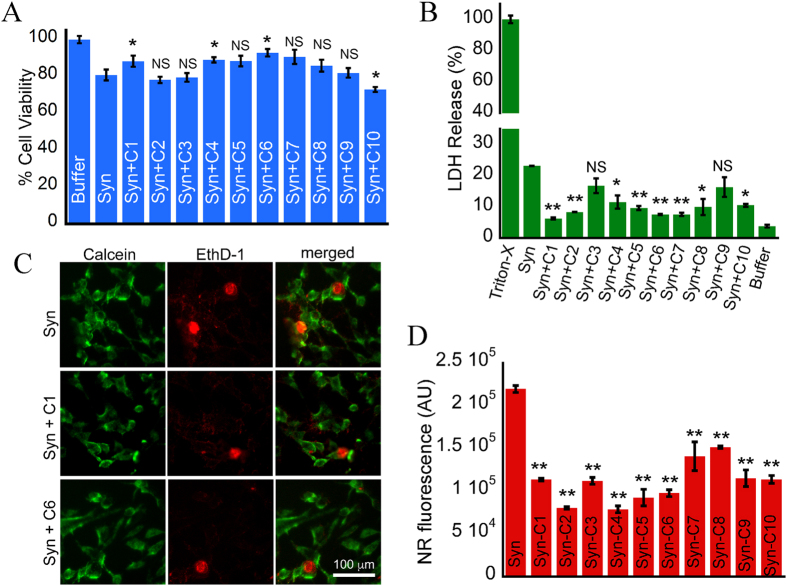
Effects of curcumin and its analogs on the toxicity and exposed hydrophobic surface of pre-formed α-Syn fibrils. (**A**) MTT assay of preformed α-Syn fibrils incubated for 48 h in absence and presence of curcumin (C1) and its analogs (C2–C10). The bar diagram of MTT assay represents mean ± SE from three independent set of experiments (for each experiment triplicate wells were used). (**B**) LDH cytotoxicity assay of preformed α-Syn fibrils incubated for 48 h in absence and presence of curcumin (C1) and its analogs (C2–C10). The bar diagram of LDH cytotoxicity assay represents mean ± SE from two independent set of experiments (for each experiment triplicate wells were used). (**C**) Calcein-AM/EthD-1 staining of SH-SY5Y cells incubated in presence of preformed α-Syn fibrils with C1 and C6. (**D**) Nile Red fluorescence of pre-formed α-Syn fibrils incubated for 48 h in absence and presence of curcumin (C1) and its analogs (C2–C10) used in this study. The bar diagram of NR fluorescence represents mean ± SE from three independent set of experiments.

Previously it has been shown that curcumin decreases the toxicity of α-Syn amyloid fibrils by reducing the exposed hydrophobic surfaces^27^. To delineate whether curcumin analogs can decrease the toxicity of preformed α-Syn fibrils, we performed cell viability assay. For this, 10-days incubated α-Syn solution was centrifuged (fibril formation was confirmed by electron microscopy (EM)) and the pelleted fibrils were then re-dissolved in same volume of 20 mM MES buffer (pH 6.0, 0.01% NaN_3_). The concentration of the fibrils was determined by subtraction of supernatant concentration from the original concentration of α-Syn kept for fibrillation. The concentration of fibrils used in this study was 80 μM, to which 40 μM curcumin analogs were added separately in microfuge tubes and kept for incubation at 37 °C for 48 h. After incubation, we measured the effect of curcumin analogs on the toxicity of preformed α-Syn amyloid fibrils by MTT reduction assay on SH-SY5Y cells. A subset of analogs (C1, C4 and C6) showed reduction in toxicity by α-Syn amyloid fibrils, where the extent of reduction was highest for C6 (92.9% cell viability) (Fig. 3A). We further performed the LDH cytotoxicity assay of preformed α-Syn fibrils incubated in absence and presence of curcumin and its analogs. Similar to the results obtained in MTT assay, we observed decrease in cell death by α-Syn fibrils incubated in presence of C1, C4 and C6 (Fig. 3B). Additionally, we also found decrease in cell death in presence of analogs C2, C5, C7, C8 and C10 in LDH assay (Fig. 3B). Moreover, the decrease in cell death by preformed α-Syn fibrils incubated in presence of C1 and C6 was further confirmed by Calcein-AM/ Ethidium homodimer-1 (EthD-1) staining assay^46^ (Fig. 3C), where in presence of C1 and C6, cells showed less nuclear staining by EthD-1, suggesting less cellular toxicity.

As change in toxicity is linked to the hydrophobic surface exposure of the amyloid fibrils and oligomers^47^,^48^, we also examined the effect of curcumin analogs on exposed hydrophobic surface of preformed α-Syn fibrils using Nile red (NR) binding assay. All curcumin analogs treated fibrils showed lesser NR fluorescence (similar to native curcumin) as compared to the untreated fibrils (Fig. 3D). The control experiments showed that curcumin analogs alone have no effect on NR fluorescence (data not shown). Further, in presence of a subset of curcumin analogs C2, C4, C5 and C6 showed less NR fluorescence for fibrils as compared to curcumin (Fig. 3D). This reduction in NR fluorescence could be due to the substituents attached to the aromatic rings in these analogs, masking the exposed hydrophobic surfaces of preformed α-Syn fibrils.

**Figure 4:**
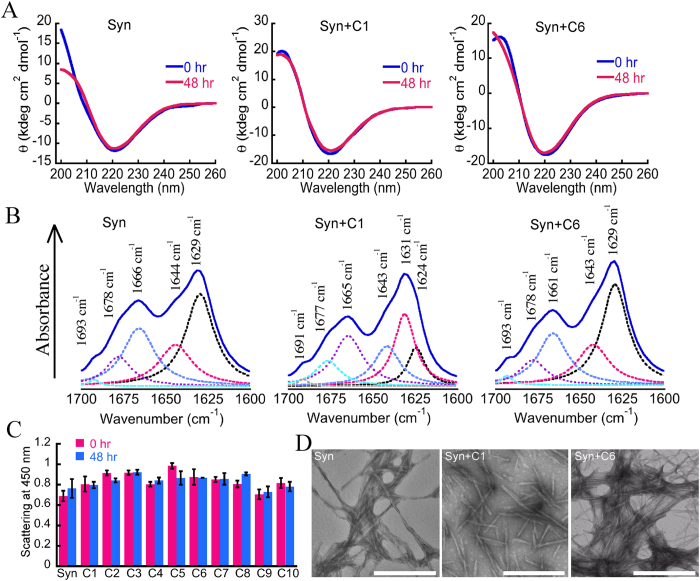
Effects of curcumin and its analogs on pre-formed α-Syn fibrils. (**A**) Secondary structure of pre-formed α-Syn fibrils incubated in absence and presence of curcumin (C1) and its analog C6 for 48 h. The secondary structure of pre-formed α-Syn fibrils remains unaltered as observed by CD. (**B**) FTIR spectra of preformed α-Syn fibrils incubated in absence and presence of curcumin (C1) and its analog C6 for 48 h showing presence of β-sheet rich structure. (**C**) The effect of curcumin analogs on size of the protein aggregates in absence and presence of curcumin (C1) and its analogs (C2–C10), as determined by static light scattering. The bar diagram of LS represents mean ± SE from four independent set of experiments. (**D**) TEM analysis to visualize the effect of curcumin and its most effective analog C6 on preformed α-Syn fibrils. Scale-500 nm.

Further, the effect of curcumin analogs on the secondary structure of protein in preformed amyloid fibrils was studied by circular dichroism (CD) spectroscopy at 0 h and after 48 h of incubation. The preformed α-Syn fibrils incubated in absence and presence of curcumin analogs showed similar extent of β-sheet rich secondary structure as evident from a negative minimum at ~218 nm in far-UV CD spectra (Figs 4A and S3). The CD data suggest that there is no major change in secondary structure components of α-Syn in the fibrils in presence of curcumin analogs even after 48 h of incubation (Figs 4A and S3). To further validate this, Fourier transform infrared spectroscopy (FTIR) was also performed with these samples, where spectra were recorded from 1500 to 1800 cm^−1^.The region characteristic of protein secondary structures (1600–1700 cm^−1^) was deconvoluted and the peaks were assigned according to published reports^49^,^50^. FTIR spectra in the 1600–1700 cm^−1^ region of preformed α-Syn fibrils in absence and presence of curcumin and its analogs are presented in Figs 4B and S4 and their percentage of secondary structures are shown in Table-S1. The α-Syn fibrils showed the intense peaks in the range of 1624 to 1638 cm^−1^, conforming β-sheet structure. Similar to the CD data, all the samples showed presence of β-sheet rich structure as characterized by the absorption peak near 1630 cm^−1^ in the FTIR spectra (Figs 4B and S4).

Next, the effect of curcumin analogs on overall size of the preformed α-Syn fibrillar aggregates was examined by static light scattering (LS) at 0 h and after 48 h of incubation. The LS data showed no significant difference in the scattering intensity between 0 h and 48 h for all samples, suggesting that curcumin and its analogs did not affect the overall size of preformed α-Syn fibrillar aggregates even after 48 h of incubation (Fig. 4C). Further, we visualized the preformed α-Syn amyloid fibrils incubated in presence and absence of curcumin analogs under transmission electron microscopy (TEM) in order to determine the possible morphological differences in fibrils (Figs 4D and S5). The electron micrographs suggest that preformed α-Syn aggregates retained their fibrillar morphology; however, variations in their lateral association were observed in presence of different curcumin analogs (Figs 4D and S5). The quantitative analysis of fibril morphology suggests that α-Syn in absence of curcumin/analogs showed fibrils with an average thickness of 16.71 ± 0.52 nm. All the samples, except C6 and C10 showed different fibril thickness as compared to α-Syn fibrils only (control) (Fig. S6A). However, the clustering was more for C6 as compared to curcumin/C1 (Fig. 4D). Maximum fibrillar thickness was seen in case of C3 (20.36 ± 0.51 nm), while in presence of C7, C8 and C9, thinner fibrils were observed (Fig. S6A). These differences in morphology could be due to rearrangement of side-chain in presence of the different curcumin analogs, without significantly affecting the protein main chain. Similar observation was reported in β-sheet intermediate (I_β_) of Aβ and its fibril counterpart, wherein protein fibrils and I_β_ despite exhibiting different morphologies and toxicity, still both showed a β-sheet secondary structure^51^. In a separate study with lysozyme, lateral association of fibrils independent of the β-sheet content under different conditions has also been reported^52^. Similarly, here we observed that curcumin/analogs binds to α-Syn fibrils and changes its appearance including lateral association/clustering without altering its secondary structural components within it.

### Effects of curcumin analogs on α-synuclein oligomers

**Figure 5:**
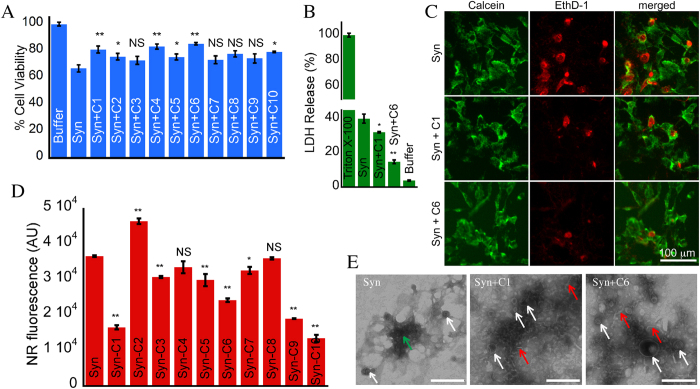
Effects of curcumin analogs on α-Syn oligomers. (**A**) MTT assay of α-Syn oligomers in absence and presence of curcumin (C1) and its analogs (C2–C10). The bar diagram of MTT assay represents mean ± SE from three independent set of experiments (for each experiment triplicate wells were used). (**B**) LDH cytotoxicity assay of α-Syn oligomers in absence and presence of curcumin (C1) and its analog C6. The bar diagram of LDH cytotoxicity assay represents mean ± SE from two independent set of experiments (for each experiment triplicate wells were used). (**C**) Calcein-AM/EthD-1 staining of SH-SY5Y cells incubated in presence of α-Syn oligomers with C1 and C6. (**D**) NR fluorescence showing different hydrophobic surface exposure of oligomers in presence of curcumin and its analogs. The bar diagram of NR fluorescence represents mean ± SE from three independent set of experiments. (**E**) Morphological analysis by EM of oligomers treated with and without curcumin and analog C6. The various morphologies are marked with arrow of different colors. White arrow represents circular, red arrow represents linear structures and green arrow represents amorphous aggregates. Scale bar is 500 nm.

Recent studies have suggested that toxicity of α-Syn oligomers are more when compared to fibrils^11^,^12^. It has been shown that α-Syn mutants that form oligomers preferentially are more toxic and kill dopaminergic neurons in animal models as compared to fibril forming mutants^12^. Therefore, compounds that effectively reduce the toxicity of oligomers could be beneficial for the treatment of PD. In the present study, we tested the effect of curcumin and its analogs on the toxicity, hydrophobic exposure and morphology of α-Syn oligomers isolated using size exclusion chromatography (SEC). The toxicity of α-Syn oligomers in presence and absence of curcumin/analogs were initially tested using MTT assay. The results of the MTT assay showed that in presence of α-Syn oligomers (10 μM), viability of SH-SY5Y cells decreased to 61% suggesting that the α-Syn oligomers are cytotoxic (Fig. 5A). However, when these oligomers were pre-incubated with curcumin and its analogs (10 μM α-Syn + 5 μM curcumin/analogs) at room temperature for 30 minutes in dark, the toxicity of α-Syn oligomers decreased significantly (except for C3, C7, C8 and C9) (Fig. 5A). The maximum cell viability was observed in case of C6 (cell viability ~85%), indicating that this could be the most effective compound in reducing oligomer toxicity. To further validate the effectiveness of C6 to reduce the cytotoxicity of α-Syn oligomers, we also performed LDH cytotoxicity assay and Calcein-AM/Ethidium homodimer-1 staining. Oligomers incubated for 30 minutes in absence and presence of C1 were used as controls. We found that, α-Syn oligomers incubated in presence of C1 and C6 showed reduced LDH release as well as less ethidium homodimer staining (Figs 5B,C). The data suggest that both curcumin (C1) and analog C6 are indeed effective in reducing the toxicity of α-Syn oligomers. Overall, our results suggest that treatment of curcumin and its analogs has the potential to reduce the toxicity of preformed α-Syn oligomers. As previously it has been proposed that curcumin reduces the oligomers toxicity by altering their exposed hydrophobic surface^27^, we also measured the hydrophobic surface exposure of preformed α-Syn oligomers incubated with curcumin and its analogs. Consistent with previous observation, preformed α-Syn oligomers (isolated from SEC^27^) incubated in presence of curcumin analogs showed significant decrease in NR fluorescence (except C4 and C8) as compared to α-Syn oligomers alone (Fig. 5D). Oligomers incubated in presence of C4 and C8 showed similar extent of NR fluorescence as that of α-Syn oligomers only. In contrast to other analogs, oligomers incubated with C2 showed increase in NR fluorescence, although, the C2 treated oligomers showed slight reduction in the toxicity (Fig. 5A,D). This result suggests that possibly C2 might have other alternative mechanism(s) for reducing the oligomer toxicity. Altogether, the toxicity and NR data on preformed fibrils and oligomers, suggest that analog C6 is the most effective neuroprotective curcumin analog among the various analogs studied here. Therefore, in the next step, we performed TEM to visualize the effect of curcumin (C1) and its most effective analog C6 on the α-Syn oligomers. The electron microscopy data showed that incubation of preformed α-Syn oligomers in presence of C1 and C6 significantly altered their morphology (Fig. 5E). α-Syn oligomers (control) showed mostly globular and some amorphous aggregates, whereas in presence of C1 or C6, the oligomers morphology converted into mostly short, curvy and circular structures (indicated by arrow). However, compared to the C1, the C6 treatment also resulted in few thinner and elongated structures of α-Syn (Fig. 5E).

### Extent of binding of curcumin analogs to oligomeric and fibrillar species of α-synuclein

**Figure 6:**
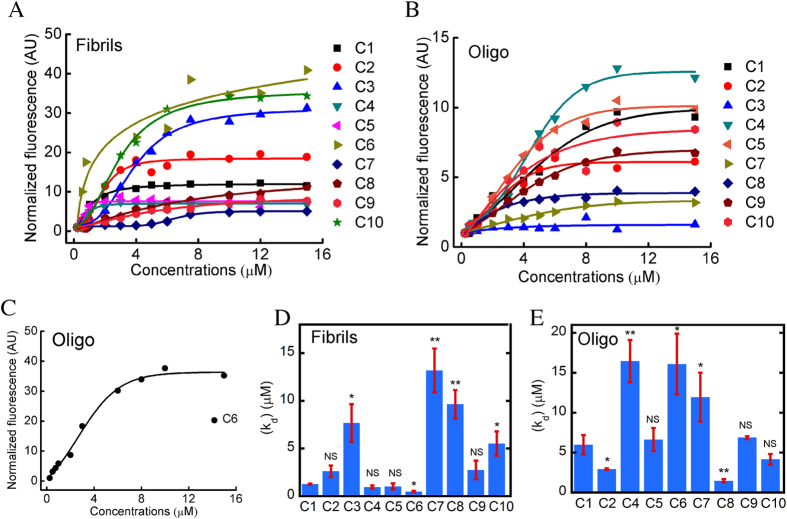
Extent of binding of curcumin analogs with fibrillar and oligomeric species of α-Syn. Saturation plots of curcumin and its analogs for α-Syn fibrils (**A**) and oligomers (**B,C**). Dissociation constants (K_d_) values of curcumin and its analogs for α-Syn fibrils (**D**) and oligomers (**E**). The bar diagram of dissociation constants represent mean ± SE from three independent set of experiments.

To estimate the comparative binding affinity of curcumin analogs to the α-Syn fibrils and oligomers, curcumin fluorescence assay was performed for each analog. For binding study with fibrils, 10 μM of α-Syn fibrils were incubated in presence of varying concentration of curcumin and its analogs (0.25 μM to 30 μM) for 30 min at RT in dark and then fluorescence spectra were recorded. The normalized fluorescence intensity of different concentrations of curcumin/analogs at their corresponding λ_max_ was plotted to obtain their respective saturation plots (Figs 6A and S7). From the saturation plot, dissociation constant (K_d_) was calculated (see method section). The K_d_ values of curcumin and its analogs for α-Syn fibrils are shown in Fig. 6D. The maximum value of K_d_ was obtained for analog C7 (13.18 ± 2.29 μM) while the minimum value was observed for analog C6 (0.482 ± 0.07 μM) (Fig. 6D and Table S2).

Similarly, we also determined the binding affinity of curcumin/analogs to α-Syn oligomers. The normalized fluorescence intensity at λ_max_ of curcumin/analogs was plotted against various concentrations and their respective saturation plots were obtained (Fig. 6B,C and S8). Among all the analogs, maximum value of K_d_ was obtained for C4 and C6 (16.46 ± 2.63 μM and 16.08 ± 3.81 μM respectively), while minimum value was observed for C8 (1.46 ± 0.20 μM). We did not observe any significant binding of C3 with α-Syn oligomers (Table S2). The dissociation constant (K_d_) of curcumin analogs for fibrils and oligomers suggested that all curcumin analogs (except C2, C7, C8 and C10) possess low affinity towards oligomers compared to fibrils. C2 and C7 showed similar affinity, while C8 and C10 possess higher binding affinity to oligomers as compared to fibrils.

In addition, we also determined the binding of curcumin and its analogs with α-Syn monomers, but none of these analogs showed significant increase in fluorescence under similar conditions (Fig. S9), indicating that binding affinity of these analogs with α-Syn monomers was negligible.

### Curcumin analogs accelerated α-synuclein aggregation

**Figure 7:**
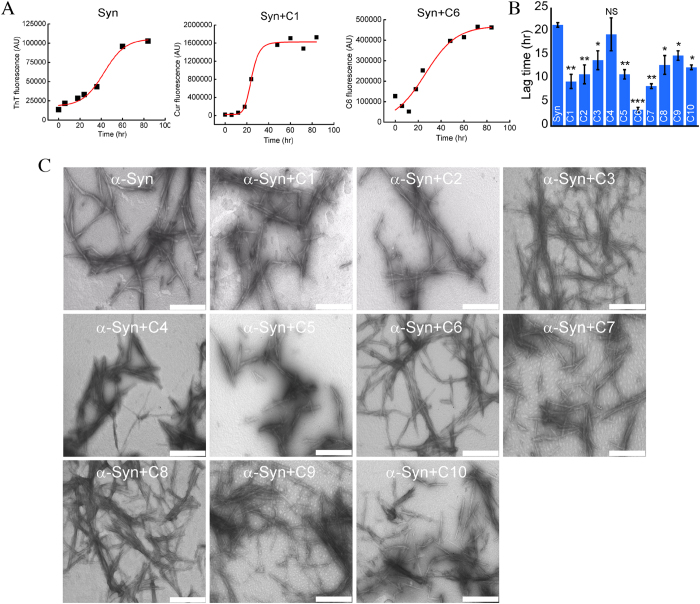
Effects of curcumin and its analogs on α-synuclein aggregation. (**A**) Aggregation kinetics of α-Syn in absence (measured by ThT fluorescence) and in presence of C1 and C6 (measured by compound fluorescence). (**B**) The lag time of aggregation for α-Syn in absence and presence of curcumin/analogs. The bar diagram of lag time represents mean ± SE from three independent set of experiments. (**C**) Morphological analysis of α-Syn aggregates formed in absence and presence of curcumin and its analogs. Scale bars are 500 nm.

Thioflavin T (ThT) dye is generally used to measure the kinetics of protein/peptides aggregation during amyloid formation^53^. However, recently it has been suggested that in presence of polyphenolic compounds like curcumin, ThT is not a reliable dye to measure the aggregation kinetics, because polyphenolic compounds interfere with the ThT fluorescence^54^. Therefore, we excluded the use of ThT fluorescence in presence of curcumin analogs. Since curcumin and its analogs have intrinsic fluorescence, which increase once they bind to oligomers and fibrils of α-Syn, we utilized this property to probe the aggregation of α-Syn in presence of curcumin analogs. ThT fluorescence was used to monitor the aggregation kinetics of α-Syn only as a control. A sigmoidal growth curve for α-Syn aggregation both in presence and absence of curcumin analogs was observed, which is a characteristic of amyloid formation (Figs 7A and S10).

Curcumin and its analogs showed increased fluorescence intensity with the intermediate and fibrillar states of the α-Syn, while none of these analogs showed substantial fluorescence at the beginning of the aggregation (Figs 7A and S10). On the basis of increase in fluorescence with time, we calculated the lag times (t_lag_)^55^ for α-Syn aggregation in absence and presence of curcumin (Fig. 7B and Table S3). The lag time of aggregation for α-Syn in presence of curcumin and its analogs showed a range, with the smallest of 3.5 ± 0.5 h for C6 and the largest of 19.5 ± 3.5 h for C4 (Fig. 7B and Table S3). As lag time is inversely related to the nucleation rate in aggregation kinetics, we found that the rate of α-Syn aggregation in presence of C6 was fastest and C4 showed slowest (Figs 7B and S10). Subsequently, we examined the effect of these analogs on α-Syn oligomerization by time dependent static light scattering experiment. Consistent with fibrillation kinetics, all analogs were found to increase the α-Syn oligomerization (Fig. S11). Additionally lag time calculated from light scattering data also showed lesser lag time of α-Syn aggregation in presence of curcumin/analogs (Table S4). Further, the secondary structure of α-Syn in presence and absence of curcumin and its analogs, at the beginning and after aggregation kinetics were monitored by CD. At the beginning of aggregation kinetics (0 h), mostly random coil conformation was observed for α-Syn in all conditions (Fig. S12). The CD spectra of α-Syn in absence and presence of curcumin and its analogs showed mostly β-sheet rich conformation at the end of aggregation study (Fig. S12). After aggregation, TEM study was done to determine the effects of these analogs on the morphology of the resultant aggregates. Visual inspection of the electron micrographs showed that α-Syn aggregates formed in presence of curcumin analogs C4, C5, C7 C9 and C10 produce fibrils with shorter length (Fig. 7C), whereas in presence of other analogs showed indistinguishable fibrillar morphology as compared to fibrils formed in absence of analogs (Fig. 7C). Interestingly, we find that the average fibril thickness in presence of C2, C4, C6 and C7 are more in comparison to fibrils formed in presence of other analogs (Fig. S6B).

**Figure 8:**
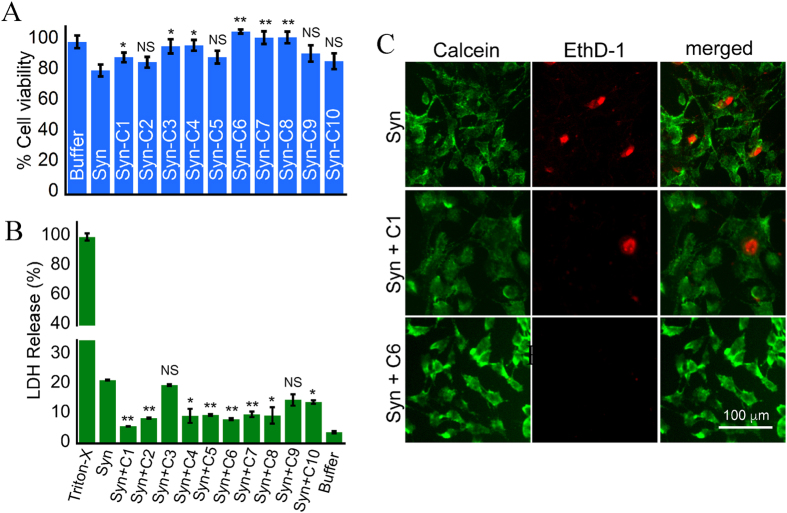
Effects of curcumin and its analogs on toxicity of α-Syn aggregation kinetics end product. (**A**) MTT assay of α-Syn fibrils formed in absence and presence of curcumin (C1) and its analogs (C2–C10). The bar diagram of MTT assay represents mean ± SE from three independent set of experiments (for each experiment triplicate wells were used). (**B**) LDH cytotoxicity assay of α-Syn fibrils formed in absence and presence of curcumin (C1) and its analog C6. The bar diagram of LDH cytotoxicity assay represents mean ± SE from two independent set of experiments (for each experiment triplicate wells were used). (**C**) Calcein-AM/EthD-1 staining of SH-SY5Y cells incubated in presence of α-Syn fibrils formed in absence and presence of C1 and C6.

Next, we measured the effect of α-Syn fibrils formed in presence and absence of curcumin analogs on the viability of SH-SY5Y neuronal cell line by MTT reduction assay. SH-SY5Y cells were incubated with 10 μM α-Syn fibril formed in presence of various curcumin analogs for 36 h. The α-Syn fibrils formed in presence of all the analogs showed reduction in toxicity as compared to α-Syn fibrils only. However, the extent of decrease in toxicity was different for different analogs. The maximum cell viability was observed for C6 (Fig. 8A). The results suggest that curcumin and its analogs have protective role in reducing the toxicity of α-Syn fibrils on SH-SY5Y neuronal cell death. Additionally, we also performed LDH cytotoxicity assay using SH-SY5Y cells to confirm these results. The result of LDH assay of α-Syn fibrils formed in presence and absence of curcumin analogs also showed reduction in toxicity as compared to α-Syn fibrils only, except for α-Syn fibrils formed in presence of C3 and C9 (Fig. 8B). The α-Syn fibrils formed in presence of C3 and C9 showed similar extent of toxicity as that of α-Syn fibrils only. From MTT reduction and LDH release assay, we observed that α-Syn fibrils formed in presence of analogs C1 and C6 showed less toxicity to cells as compared to α-Syn fibrils alone. The calcein-AM/ethidium homodimer-1 staining also further corroborated the results obtained from MTT and LDH assay (Fig. 8C).

## Discussion

The α-Syn oligomers and fibrils are believed to be toxic species responsible for the PD pathogenesis. Recent studies have suggested that toxicity of oligomers are more compared to that of fibrils^11^,^12^. The α-Syn mutant that preferentially forms oligomers showed more toxicity and dopaminergic cell death in rat model of PD compared to the wild-type α-Syn, which preferentially formed fibrils^12^. Therefore, inhibiting oligomerization and/or accelerating toxic oligomers to fibrils conversion could be effective therapeutic approach. Many recent studies have suggested that compounds that modulate the α-Syn aggregation, where toxic population of oligomers could be reduced may hold promise for drug development against PD^13^,^14^,^15^. In this context, curcumin has shown to be a promising therapeutic agent against various human diseases such as diabetes, cardiovascular disorders, cancers and neurological disorders^22^. Curcumin has antioxidant property, hence detoxify the reactive oxygen species (ROS), as well as it can also induce neurogenesis *in vivo*^56^. Previous studies have suggested that curcumin inhibits the accumulation and/or aggregation of many amyloidogenic proteins including α-Syn associated with neurodegeneration^25^,^26^,^57^,^58^. It has been shown that kinetics of α-Syn is very sensitive to experimental conditions that include buffer, pH, temperature, presence of additives and co-solvents^59^,^60^,^61^. Previously, Galvin and coworkers showed decrease in α-Syn aggregation in presence of curcumin where the authors have used 1 mM FeCl_3_ in 20 mM Tris-Cl, pH 7.5. Later, Lapidus and coworkers also showed that α-Syn aggregation and oligomerization is inhibited in presence of curcumin in 25 mM phosphate buffer of pH 7.4, 150 mM NaCl and 1 mM TCEP. In their study, the molar ratio of protein to curcumin used was 1:1.5. Additionally, the oligomerization study was done in 25 mM phosphate buffer of pH 7.4, 150 mM NaCl and 1 mM TCEP containing 10% (v/v) TFE. In contrast, in the present manuscript, we report faster aggregation kinetics of wild type α-Syn in presence of curcumin/analogs. This discrepancy could be due to different experimental conditions used in these studies. In our present work, we performed α-Syn aggregation kinetics in 20 mM MES buffer, pH 6.0 and the protein to curcumin/analogs molar ratio was 1:0.5. Moreover, in the earlier studies, the purification method and concentration of α-syn used were also different. Lapidus and coworkers used 48 μM protein for aggregation studies and 5 μM protein for oligomerization studies. Pandey *et al*. used 5 μM α-Syn protein in presence of 1 mM FeCl_3_ in their studies. In the present study, we used 300 μM α-Syn protein in the low molecular weight form^27^. Further the different experimental condition including pH may also affect the degradation rate and degradation products of curcumin, which may affect the aggregation kinetics of α-Syn^42^. We therefore conclude that the difference in observation that curcumin accelerates α-Syn aggregation rather inhibiting it, as shown in the previous studies^57^,^58^ could be due to the differences in protein purification methods, pH, buffer composition and molar ratio of protein to curcumin/analogs. Also many of the earlier studies used ThT fluorescence for monitoring α-Syn aggregation kinetics in presence of curcumin. As the fluorescence spectra of ThT and curcumin overlap with each other^54^, ThT is not a suitable dye to monitor amyloid aggregation kinetics in presence of polyphenols. Similar conflicting results were also observed in case of curcumin mediated *Aβ* aggregation. Yang *et al*. showed *in vivo* inhibition of Aβ oligomerization and fibril formation in presence of curcumin^25^. It has also been reported that the dietary supplementation of curcumin decreased the accumulation of amyloid-β (Aβ) peptide in transgenic mouse model of AD and it prevents Aβ aggregation^24^. However, recently it has been shown that curcumin accelerates amyloid-β (Aβ) fibrillation and reduces its toxicity in *Drosophila* model^62^. Additionally, our previous study suggested that curcumin accelerates fibrillation of α-Syn by binding to oligomers and reduced the toxicity of α-Syn species^27^. The major constraint for therapeutic application of curcumin is its poor stability at physiological pH and its high rate of metabolism^42^,^43^ *in vivo*. It has been shown that administration of curcumin in mouse at 0.1 g/kg dose via i.p. (intra peritoneal) route allows detecting only trace amount of curcumin (0.4 μg/g) in brain tissue^63^, suggesting its lower blood-brain barrier crossing ability. Curcumin undergoes substantial reduction through alcohol de-hydrogenase, followed by sulfation and glucuronidation as soon as it is absorbed in various tissues. Major biliary metabolites of curcumin were found to be as glucuronides of tetrahydro-curcumin and hexahydro-curcumin whereas minor biliary metabolites of curcumin include dihydroferulic acid and ferulic acid^42^. The metabolic products of curcumin were found to be less effective compared to their parental form, making it less bioactive^42^.

To overcome this limitation, multiple approaches like discovery of various natural curcumin analogs, synthesis of artificial curcumin analogs and formulation of curcumin with different oils, liposomes and nanoparticles are being executed^64^,^65^,^66^. Recently Orlando *et al.,* have screened and analyzed a library of artificial curcumin analogs that inhibited Aβ oligomerization either equal to or more than the effect of curcumin^28^. We selected nine analogs of curcumin such that they are structurally similar to curcumin, but have different functional group and could have potential effect on α-Syn aggregation. The effect of all these analogs were tested on different α-Syn species formed during aggregation process. Curcumin and its analogs when added to α-Syn displayed varied effects on amyloid formation kinetics. This could depend on the different functional groups present in each analog. The less significant effect of C8 and C9 on α-Syn aggregation might be due to the presence of two hetero nuclear aromatic rings, thiophene (C8) and pyridine (C9) indicating that benzene rings of curcumin play an essential role in modulating α-Syn aggregation. As opposed to this, an increase in aggregation rate of α-Syn was observed in presence of analogs having benzene ring in comparison to α-Syn alone (Fig. 7B). Interestingly, analog C4 has two −OMe groups present at the meta-position with respect to each other, which show lesser effect on aggregation kinetics, whereas C5 bearing same group at the para-position increases the rate of α-Syn aggregation. The data clearly indicate that the positioning of methoxy group in the aromatic ring of C4 and C5 might influence its interaction with α-Syn, thereby displaying differential effect on aggregation kinetics (Figs 7B and S10). The position effect (ortho-, meta- or para-) of substituents in the aryl rings of curcumin has previously been shown to influence their inhibitory activity on Aβ aggregation^28^. Although C3 contains three −OMe groups (which makes it highly hydrophobic in nature), it shows only minimal effect on the aggregation kinetics of α-Syn. The probable reason for this behavior of C3 analog could be due to the steric hindrance, leading to its weaker interaction with α-Syn.

Among all the curcumin analogs used, the analog C6 significantly accelerated α-Syn aggregation kinetics (lag time 3.5 ± 0.5 h) (Fig. 7B). The availability of −OCH_2_Ph substituents on either end of the molecule (Fig. 1) could play an important role in interactions between the compound and the aromatic/hydrophobic side chains of the protein, which might further allow rapid aggregation of α-Syn. In contrast, several previous studies have shown the inhibitory effect of different phenyl group bearing compounds^67^,^68^,^69^.

In the various amyloid-related diseases, the oligomers as well as fibrils were reported to act as cytotoxic species^11^,^12^. In line with these observations, several research groups have studied the effect of small molecule inhibitors on preformed aggregates and oligomers in detail^14^,^16^,^17^,^18^,^19^. Previous studies showed that these molecules bind to pre-fibrillar aggregates, oligomers or fibrils and caused destabilization of aggregates or alters the amyloidogenic pathway^27^,^70^,^71^,^72^. In the current study, when the effect of curcumin and its analogs was tested on the preformed fibrils of α-Syn, it was found that none of these compounds affected the major protein secondary structure (β-sheet) within fibrils, as determined by CD or FTIR (Figs S3 and S4), however, effectively decreased the exposed hydrophobic surface on the fibrils (Fig. 3D). It has been shown that toxicity is well correlated with exposed hydrophobic surfaces of protein aggregates^48^. We propose that curcumin and its analogs may bind and reduce exposed hydrophobic surfaces of oligomers and fibrils thereby reducing their cellular toxicity. In line with this, we performed NR binding of α-Syn fibrils and oligomers in presence and absence of curcumin analogs. Our results suggest that in presence of most of the curcumin analogs, the NR fluorescence of α-Syn oligomers and fibrils decreased. The data suggest that in presence of most of the curcumin analogs the exposed hydrophobic surfaces of both α-Syn oligomers and fibrils are reduced with varying degrees. Therefore these compounds are also expected to reduce the interaction of oligomers and fibrils with cell membrane and reduce the toxicity. Indeed, we observed less toxicity of oligomers and fibrils in presence of most of the curcumin analogs (Figs 3 and 5). From our electron microscopy studies, we further find that the addition of C1 and C6 allowed formation of morphologically different oligomers (Fig. 5E). This was further reflected from the reduced cytotoxicity observed in case of oligomers added with curcumin analogs, when compared to α-Syn oligomers only (Fig. 5A).

We further analyzed the binding of curcumin/analogs to fibrils and oligomers using compound fluorescence intensity as a parameter. We found that different analogs bound to these species to a different extent. The highest binding of analog C6 with the preformed fibrils could be due to the presence of −OCH_2_Ph groups, where phenyl group (Ph) may interact with hydrophobic grooves of fibrils. On the other hand, C7 showed very weak binding due to absence of substituent groups on the aromatic rings. The remaining analogs (except C9) showed intermediate extent of binding due to presence of moderate π-electron clouds on the aromatic rings. These electron clouds could establish weak hydrophobic interactions between curcumin analogs and the hydrophobic patches of α-Syn fibrils. The analog C9 did not show any significant binding with the preformed α-Syn fibrils, which might be due to decrease in conjugated π-electron clouds, created by the electron withdrawing N-atom in the aromatic ring (Fig. 1). Consistent with the previous finding that curcumin binds to α-Syn oligomers to a lesser extent compared to fibrils^27^, we also found that curcumin and its analogs (except C8 and C10) showed lesser binding with the oligomers compared to fibrils. Interestingly, the increased binding of analog C6 with the preformed fibrils and its highest reduction in oligomers and fibrils-mediated toxicity suggests that curcumin analog C6 may hold promise as potential therapeutic agent against PD.

## Methods

### Chemicals and reagent

All the chemicals required for the synthesis of curcumin and its analogs were of reagent grade and purchased from Spectrochem (Mumbai, India) and Sigma Aldrich (St. Louis, MO, USA). Curcumin and its analogs were prepared according to literature methods^31^. Other chemicals used in this study were purchased from Sigma Aldrich (St. Louis, MO, USA) unless mentioned specifically. Nile red was purchased from Invitrogen (Carlsbad, CA, USA). Water was double distilled and deionised using a Milli-Q system (Millipore Corp., Bedford, MA, USA).

### Preparation of stock solutions of curcumin analogs

40 mM curcumin and stock solutions of its analogs were prepared in dimethyl sulfoxide (DMSO). These stock solutions were further diluted in 20 mM MES buffer, pH 6.0, with 0.01% sodium azide. For binding affinity experiment, different stock solutions of curcumin and its analogs were freshly prepared in DMSO and further diluted in the same buffer. For each experiment the freshly prepared DMSO stocks were used.

### Spectroscopic studies of curcumin analogs

For the determination of excitation wavelengths, 40 μM of all the analogs were prepared in 20 mM MES buffer (pH 6.0, 0.01% NaN_3_), and the absorption spectra were acquired in the range of 300–600 mm in JASCO V-650 spectrophotometer. For emission wavelength determination, 50 μM of all the analogs were prepared in 20 mM MES buffer (pH 6.0, 0.01% NaN_3_), and the solutions were excited at the wavelength corresponding to maximum absorption (Fig. S1), and emission spectra were acquired up to 650 nm. The spectra were monitored by using FluoroMax-4 spectrofluorometer (HORIBA JobinYvon) with both excitation and emission slit widths of 5 nm.

### Stability of curcumin analogs

40 mM stock solutions of curcumin and its analogs were prepared in DMSO. 1μl of this DMSO-stock solution was transferred to 2 ml microfuge tube and 1 ml of 20 mM MES buffer (pH 6.0, 0.01% NaN_3_) was added to it and mixed immediately by vortexing. These solutions were incubated at 37 ^o^C and their respective fluorescence was taken at certain time intervals. The fluorescence was monitored by placing the 200 μl of the incubated solution in 0.1 cm path-length quartz cuvette (Hellma, Forest Hills, NY) and the spectra were acquired by using FluoroMax-4 spectrofluorometer (HORIBA JobinYvon) instrument with excitation and emission slit widths of 5 nm and 10 nm respectively. The fluorescence intensity maxima for each sample were plotted against time of incubation and fitted in exponential decay 1 in the Origin 8.0 software according to manufacture’s protocol (Fig. 2A).

### MTT reduction assay

SH-SY5Y cells (neuroblastoma cell line) were cultured in DMEM (Dulbecco’s modified Eagle’s medium) with 10% FBS, in a 5% CO_2_ incubator at 37 °C. Cells were seeded at a density of 10,000 cells/well in 96-well plates. After 20 h of incubation fresh media containing curcumin analogs and different species of α-Syn (fibrils and oligomers) incubated with curcumin analogs at a concentration of 2:1 (10 μM of α-Syn and 5 μM curcumin analogs) were added. Cells were further incubated for 36 h at 37 °C. For control, media containing buffer with DMSO (present in samples) were used as controls. After 36 h of incubation, 10 μl of MTT solution (5 mg/ml prepared in PBS) was added to each well and incubated for 4 h. Subsequently, 100 μl of SDS-DMF solution (50% DMF and 20% SDS, pH 4.75) were added and kept for overnight incubation. The absorption values at 560 nm and 690 nm (background absorbance) were determined using a SpectraMax M2e microplate reader (Molecular Devices, USA). The cell viability was plotted as percentage cell viability by considering 100% viability for the buffer control.

### Lactate Dehydrogenase (LDH) release assay

10 μM of α-Syn oligomers/fibrils incubated with and without 5 μM curcumin/analogs were added to SH-SY5Y cells (10,000 cells/well) in 96 well plate. After 36 h of incubation with samples the LDH release assay was performed using LDH toxicological kit (TOX- 7, Sigma) according to the manufacturer’s instructions. The absorption values at 490 nm and 690 nm were determined using a SpectraMax M2e microplate reader (Molecular Devices, USA). The percent LDH release was plotted as difference in the absorbance value of 490 nm and 690 nm, considering 100% release for the TritonX-100 treated cells.

### Calcein-AM/Ethidium homodimer-1 live dead assay

For this assay, SH-SY5Y cells were seeded in 96 well plate and incubated with identical concentrations of oligomers/fibrils (as used in MTT assay) in absence and presence of curcumin and its analogs for 36 h in 5% CO_2_ incubator at 37 ^o^C. After incubation, the media was taken out and cells were washed twice with DPBS (Dulbecco’s PBS) to remove residual serum and samples. Cells were then incubated in presence of 1 μM Calcein-AM and 1 μM Ethidium homodimer-1 for 20 min at room temperature in dark. Calcein-AM/Ethidium homodimer-1 signal was then checked and imaged with an inverted fluorescence microscope (Leica DMi8). Calcein-AM is a dye which is cleaved to calcein by the esterase of live cells when they permeate the cell. The free calcein emits strong fluorescence signal at ~535 nm, which could be easily observed in the green channel of a microscope. EthD-1 is permeable to dead cells, which have an altered membrane structure. Once it enters a dead or dying cell, it binds with the nucleus emitting strong fluorescence at ~617 nm, which could be observed in the red channel.

### Protein purification

α-Syn protein was expressed in *Escherichia coli* BL21 strain in accordance with the protocol described by Volles *et al*.^73^ with slight modifications as described previously^27^,^74^.

### Preparation of low molecular weight (LMW) α-Syn

The lyophilized powder of α-Syn (stored at −20 ^o^C) was dissolved at a concentration of 20 mg/ml in 20 mM MES buffer, pH 6.0, containing 0.01% sodium azide. Few microliters of 2 M NaOH solution was added and mixed thoroughly until clear solution appeared. The final pH was adjusted to 6.0 after addition of few μl of 2N HCl. The protein solution was dialyzed overnight at at 4 ^o^C in the same buffer in order to remove salt and fragmented proteins/peptides using a 10 kDa MWCO mini-dialysis unit, which were pre-washed for 30 min in the milli-Q water (Millipore, USA). Furthermore, the larger aggregates (if any), were removed by centricon YM-100 MWCO filter centrifuge unit (Millipore, USA). The resulting flow through solution contained LMW species of less than 100 kDa, was collected and used for the study. Concentration of LMW was measured by UV absorbance spectra at 280 nm, considering epsilon value of α-Syn as 5960 M^−1^ cm^−1^.

### Amyloid fibril formation

The α-Syn amyloid aggregation was started with LMW α-Syn of concentration ~300 μM in 1.5 ml microfuge tube in 20 mM MES buffer, pH 6.0, with 0.01% NaN_3_. To study the effect of curcumin on fibril formation by α-Syn, 80 μM of α-Syn in presence and absence of 40 μM of curcumin and its analogs were incubated. The microfuge tubes with protein solutions were placed into Echo-Therm model RT11 vertical rotor (Torrey Pines Scientific, USA) with a speed of 50 rpm, at 37 ^o^C inside an incubator. The fibril formation was regularly monitored by ThT binding, curcumin fluorescence, CD and confirmed by EM after fibrillation. In order to know the effect of curcumin analogs on preformed fibrils and for binding affinity study, the α-Syn fibrils were prepared according to the above protocols.

### Circular dichroism spectroscopy (CD)

20 μl of protein solution from 80 μM stock was diluted to 200 μl in 20 mM MES buffer, pH 6.0 with 0.01% NaN_3_ such that final protein concentration becomes 8 μM. The protein solution was then placed into a quartz cell (Hellma, Forest Hills, NY) of 0.1 cm path-length. Spectra were recorded using JASCO-810 instrument at 25 ^o^C. Spectra acquisition was over the wavelength range of 198–260 nm with the scan speed of 100 nm/min, bandwidth 1 nm, data pitch 1 nm and response time of 1 sec. Processing of raw data was done by subtraction of buffer spectra and subsequently smoothing according to the manufacturer’s instructions.

### Fourier Transformed Infra Red Spectroscopy (FTIR)

For FTIR experiments, small amount of KBr was taken in a mortar and ground with the pestle to make fine powder. KBr powder was compressed at the pressure of ~5 ton with a hydraulic pressure pump to make pellet. The pellet was placed under IR lamp and 10 μl of 80 μM protein solutions (α-Syn fibrils and α-Syn fibrils with curcumin analogs) were spotted and dried immediately. For baseline correction, 10 μl of the corresponding buffer (MES, pH 6.0, 0.01% NaN_3_) spotted on another KBr pellet was used. The FTIR spectra were recorded in the range of 1800–1500 cm^−1^ at a resolution of 4 cm^−1^. An average of 32 scans was recorded using BrukerVertex-80 instrument with DTGS detector. The FTIR spectra corresponding to amide-I region (1700–1600 cm^−1^) were used for secondary structural analysis. The acquired spectra was processed via Fourier Self Deconvolution (FSD), Lorenzian curve fitting and integration procedure using OPUS-65 software. The various peaks in the curve fitted spectra were assigned in accordance with published reports^49^,^50^. From these peaks, the percentage of various secondary structures for each of these samples was calculated.

### Curcumin analogs fluorescence

The fluorescence spectra of curcumin analogs in presence and absence of α-Syn (10 μM of α-Syn and 5 μM curcumin/analogs) were acquired by exciting the samples at their corresponding absorption maxima. The experiments were performed in FluoroMax-4 spectrofluorometer (HORIBA JobinYvon) with excitation and emission slit widths of 5 nm. Fluorescence was done in 20 mM MES buffer (pH 6.0, 0.01% NaN_3_). On the basis of increase in fluorescence with α-Syn aggregation, the lag times (t_lag_) for α-Syn aggregation in absence and presence of curcumin analogs were calculated^55^ using the equations (1) and (2)


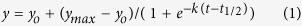



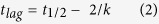


where, “y” is the intensity of ThT/curcumin analogs fluorescence at time “t” while “y_0_” is the fluorescence intensity at initial time point^55^.

### Static light scattering

Effect of curcumin analogs on the preformed α-Syn fibrils was monitored by the static light scattering. To do so, 200 μl of preformed α-Syn fibrils incubated with curcumin analogs at a concentration of 2:1 (10 μM preformed α-Syn and 5 μM curcumin analogs) was taken in aquartz cuvette of 0.1 cm path-length (Hellma, Forest Hills, NY). The scattering of light was measured using both excitation and emission wavelengths at 450 nm for 60 sec with 2 nm slit width. FluoroMax-4 spectrofluorometer (HORIBA JobinYvon) was used for this scattering study. The intensity of scattered light was plotted against incubation time (h).

### Nile red (NR) binding assay

Stock solution of NR was prepared in DMSO (1 mM). To study the effect of curcumin analogs on preformed α-Syn fibrils and oligomers (obtained from SEC), 0.2 μl of 1 mM NR was added to 200 μl of the 10 μM α-Syn fibrils, prepared in presence and absence of curcumin analogs. For NR fluorescence measurement, samples were incubated with NR for 5 min and excited at 550 nm and the emission spectra were recorded in the range of 565–720 nm. Similar experimental protocol was used for NR binding to oligomers. FluoroMax-4 spectrofluorometer (HORIBA JobinYvon) was used with excitation and emission slit widths of 5 nm. The maximum NR fluorescence obtained at ~630 nm was used for plotting bar diagrams.

### Transmission Electron Microscopy

5 μl of various species of α-Syn (50 μM) incubated in absence and presence of curcumin and its analogs were spotted on carbon coated formavar grid and air dried for 10 min. The samples were then washed thrice with autoclaved MQ. The samples were then stained with 10 μl of uranyl formate 1% (w/v) and incubated in dark for 10 min. The EM image of each sample was acquired at 120 kV with magnifications in the range of 26,000X and 43,000X using a transmission electron microscope (TECNAI12 D312 FEI, Netherlands).

### Isolation of α-Syn oligomers from size exclusion chromatography

For isolation of α-Syn oligomers, we used previously established protocol^27^. Briefly, lyophilized α-Syn powder was dissolved in MES buffer, pH 6.0, 0.01% NaN_3_, at a concentration of 50 mg/ml. The dissolved protein was then centrifuged at 14,000 × g for 30 min at 4 ^o^C using a table top microcentrifuge (HITACHI, himac CT15RE, Japan). The supernatant was taken out and injected into a S200-Superdex gel filtration column attached with AKTA purifier (GE healthcare). The protein was eluted out at 4 ^o^C in MES buffer at a flow rate of 0.2 ml/min. Fractions of 200 μl were collected. α-Syn oligomers were eluted at ~8 ml (void volume) and used for further study.

### Determination of binding affinity by curcumin analogs fluorescence

The binding of curcumin/analogs to α-Syn fibrils were determined based on change in fluorescence intensity. For this study, 10 μM α-Syn fibrils were incubated in presence and absence of varying concentration of curcumin/analogs (0.25 μM to 30 μM) for 30 min at RT in dark. Immediately after incubation, fluorescence study was performed (for the excitation and emission ranges for each compounds, see Fig. S1). Experiments were performed using FluoroMax-4 spectrofluorometer (HORIBA JobinYvon) with excitation and emission slit widths of 5 nm. The maximum fluorescence intensity for curcumin/analogs was normalized with respect to that of the lowest concentration of each analog. The normalized fluorescence intensity was plotted against increasing concentration of curcumin/analogs and the dissociation constant was determined using the equation


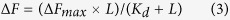


where, “Δ*F*” is the change in the fluorescence intensity of curcumin/analogs, “Δ*F*_max_” is the change in the fluorescence intensity at saturation point and “*L*”is the concentration of curcumin/analogs. The data were analyzed in Graphpad Prism software according to established method as used previously^74^. Similarly, the dissociation constant of curcumin and its analogs with preformed α-Syn oligomers and monomers (obtained from SEC) were also determined.

### Statistical analysis

The statistical significance was calculated by one-way ANOVA and subsequently by Student-Newman-Keuls Multiple Comparison post hoc test, *P < 0.05, **P < 0.01, ***P < 0.001; NS P > 0.05.

## Additional Information

**How to cite this article**: Jha, N. N. *et al*. Effect of curcumin analogs on α-synuclein aggregation and cytotoxicity. *Sci. Rep.* **6**, 28511; doi: 10.1038/srep28511 (2016).

## Supplementary Material

Supplementary Information
