# Supplementary material for: Effect of curcumin analogs onα-synuclein aggregation and cytotoxicity
Source: Sci Rep. 2016 Jun 24;6:28511. doi: 10.1038/srep28511 (PMC4919791; doi:10.1038/srep28511)
Supplement: Supplementary Information [file srep28511-s1.pdf]

Supplementary Information

Effect of curcumin analogs on α-synuclein aggregation and cytotoxicity

Narendra Nath Jha[1], Dhiman Ghosh[1], Subhadeep Das[1,3], Arunagiri Anoop[1], Reeba S. Jacob[1], Pradeep K. Singh[1], Narasimham Ayyagari[2], Irishi N. N. Namboothiri[2] and Samir K. Maji[1]*

[1] Department of Biosciences and Bioengineering, Indian Institute of Technology Bombay, Mumbai 400 076, India

[2] Department of Chemistry, Indian Institute of Technology Bombay, Mumbai 400 076, India

[3] IITB Monash Research Academy, Indian Institute of Technology Bombay, Mumbai 400 076, India.

* To whom correspondence should be addressed. Email: samirmaji@iitb.ac.in

Supplementary Results.

Spectroscopic properties of Curcumin analogs. To characterize the spectral properties of curcumin analogs, all the analogs were dissolved in dimethylsulfoxide (DMSO) and then diluted in 20 mM 2-(N-morpholino)ethanesulfonic acid (MES) buffer, pH 6.0. For determining absorption property, the UV absorption spectra of all the curcumin analogs (40 μM) were recorded in the range of 300-600 nm. The analogs showed absorption maxima within the wavelength range of 382 nm to 438 nm (Fig. S1A and S1B). The absorption spectra of analogs C3, C6, C7, C8, C9 and C10 showed blue shift in the wavelength maxima as compared to curcumin (Fig. S1). However, C4 and C5 showed red shift as compared to curcumin, suggesting that difference in the absorption maxima of these analogs might be due to different substituents attached to the aromatic rings (Fig. 1). For fluorescence properties determination, 50 μM of all the curcumin analogs were prepared in 20 mM MES buffer, pH 6.0. Then each curcumin analog was excited at their maximum absorption wavelength and the emission spectra were recorded. When excited at 425 nm, curcumin gives fluorescence maximum at 533 nm. However, each curcumin analog showed different fluorescence spectra with difference in their fluorescence emission intensity (Fig. S1). A subset of analogs, C2, C4, C6 and C10 showed more intense fluorescence signal (Fig. S1B). This increase in fluorescence intensity could be due to presence of electron donating groups (-OMe and -OCH_2Ph) on the aromatic rings. Although -OMe group is present in curcumin (C1), it showed lesser fluorescence intensity than C2, C4, C6 and C10. This lesser fluorescence intensity in C1 could be due to the –meta position of –OMe group that is less effective as electron donating group. The remaining analogs (C3, C5, C7, C8 and C9) showed lesser fluorescence intensity as compared to curcumin. In case of C3, decrease in fluorescence intensity could be due to the presence of three -OMe groups close to each other that may lead to steric hindrance, affecting its conjugation and thereby decreasing the fluorescence intensity. In C5, one of the two –OMe group is present at -meta position in each aromatic ring that is less effective as electron donating group. Additionally the other moderately bulky –OMe group is present at the -ortho positions with respect to the planer backbone, which may also cause steric hindrance to some extent and thereby reducing the electron donating capacity. Analog C7 lacks the electron

donating group (-OMe) attached with the aromatic ring, hence shows decreased fluorescence intensity. On the other hand, analogs C8 and C9 containing electron withdrawing atoms (S and N; Fig. 1) in the aromatic rings which in turn lead to decrease in conjugated π-electron clouds, and hence decrease in fluorescence intensity (Fig. S1).

Supplementary Figure Caption.

Fig. S1 Absorbance (black) and fluorescence (red) spectra of curcumin and its analogs showing different excitation and emission wavelength maxima (A). The absorbance and fluorescence intensity maxima were listed in tabular form (B).

Fig. S2 Fluorescence intensity of curcumin and its analogs during incubation. The plots of normalized fluorescence intensity of each curcumin analogs showing decrease in fluorescence intensity with time. The slope of C2 and C4 are steeper than curcumin, suggest that C2 and C4 are less stable than curcumin while other analogs with reduced slopes are more stable than curcumin under similar conditions.

Fig. S3 Secondary structure of pre-formed α-Syn fibrils incubated in absence and presence of curcumin and its analogs monitored by CD at 0 h and 48 h.

Fig. S4 FTIR spectra of α-Syn fibrils incubated in absence and presence of curcumin and its analogs after 48 h, showing presence of β-sheet rich structure.

Fig. S5 Electron micrographs of pre-formed α-Syn fibrils incubated in presence of curcumin (C1) and its analogs (C2 to C10). Scale-500 nm

Fig. S6 Average fibril thickness of α-Syn preformed fibrils after 48 h of incubation in absence and presence of curcumin and its analogs (A) and fibrils formed in absence and presence of curcumin and its analogs (B). The bar diagrams of fibril thickness represent mean±SE from twenty different filaments/fibrils.

Fig. S7 Fluorescence intensity maxima of curcumin and its analogs after mixing with α-Syn fibril showing different extent of binding by different analogs compared to curcumin.

Fig. S8 Fluorescence intensity maxima of curcumin and its analogs after mixing with α-Syn oligomers showing different extent of binding by different analogs compared to curcumin.

Fig. S9 Fluorescence spectra of curcumin and its analogs incubated with monomeric species of α-Syn. No significant differences in fluorescence intensity of 10 μM curcumin or its analogs in buffer (10) and in presence of 10 μM α-Syn monomers (AS-10).

Fig. S10 Kinetics of α-Syn aggregation. In presence of curcumin and its analogs, kinetics of aggregation was monitored as increase in compound fluorescence with time. For α-Syn only (control), the aggregation was measured by increase in ThT fluorescence with time.

Fig. S11 Kinetics of α-Syn oligomerization/aggregation monitored by increase in static light scattering intensity of α-Syn in absence and presence of various curcumin and its analogs.

Fig. S12 Secondary structure determination of α-Syn in presence and absence of curcumin and its analogs at the beginning and end of aggregation kinetics monitored by CD.

Supplementary Figures

Fig. S1

A

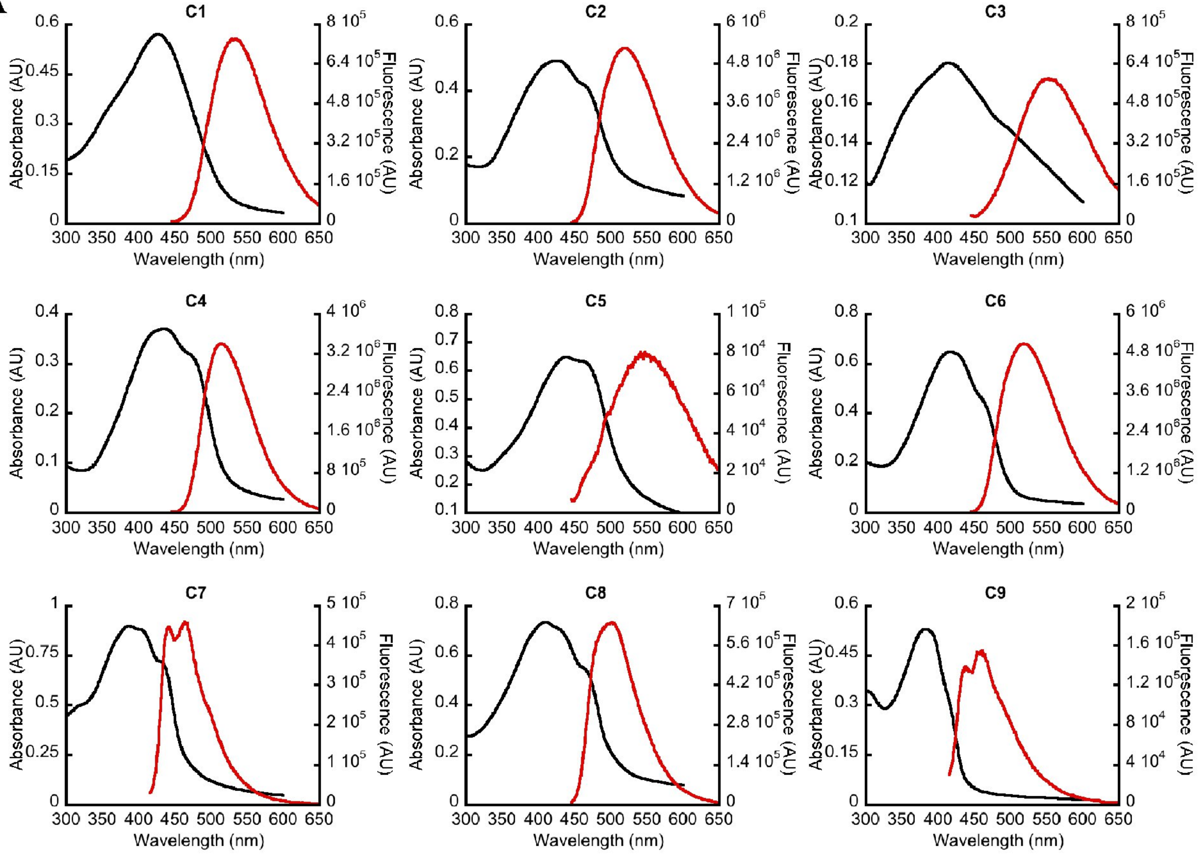

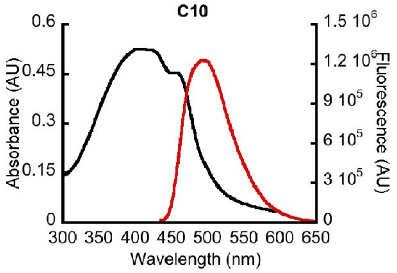

B

Curcumin Analogs	Absorbance Maxima (nm)	Fluorescence Maxima (nm)	Maximum Flu. Intensity (AU)
C1	425	533	741950
C2	425	521	5288510
C3	415	552	582550
C4	435	514	3405600
C5	438	547	81260
C6	415	518	5110460
C7	385	463	459820
C8	410	502	642040
C9	382	459	154010
C10	405	494	1225090

Fig. S2

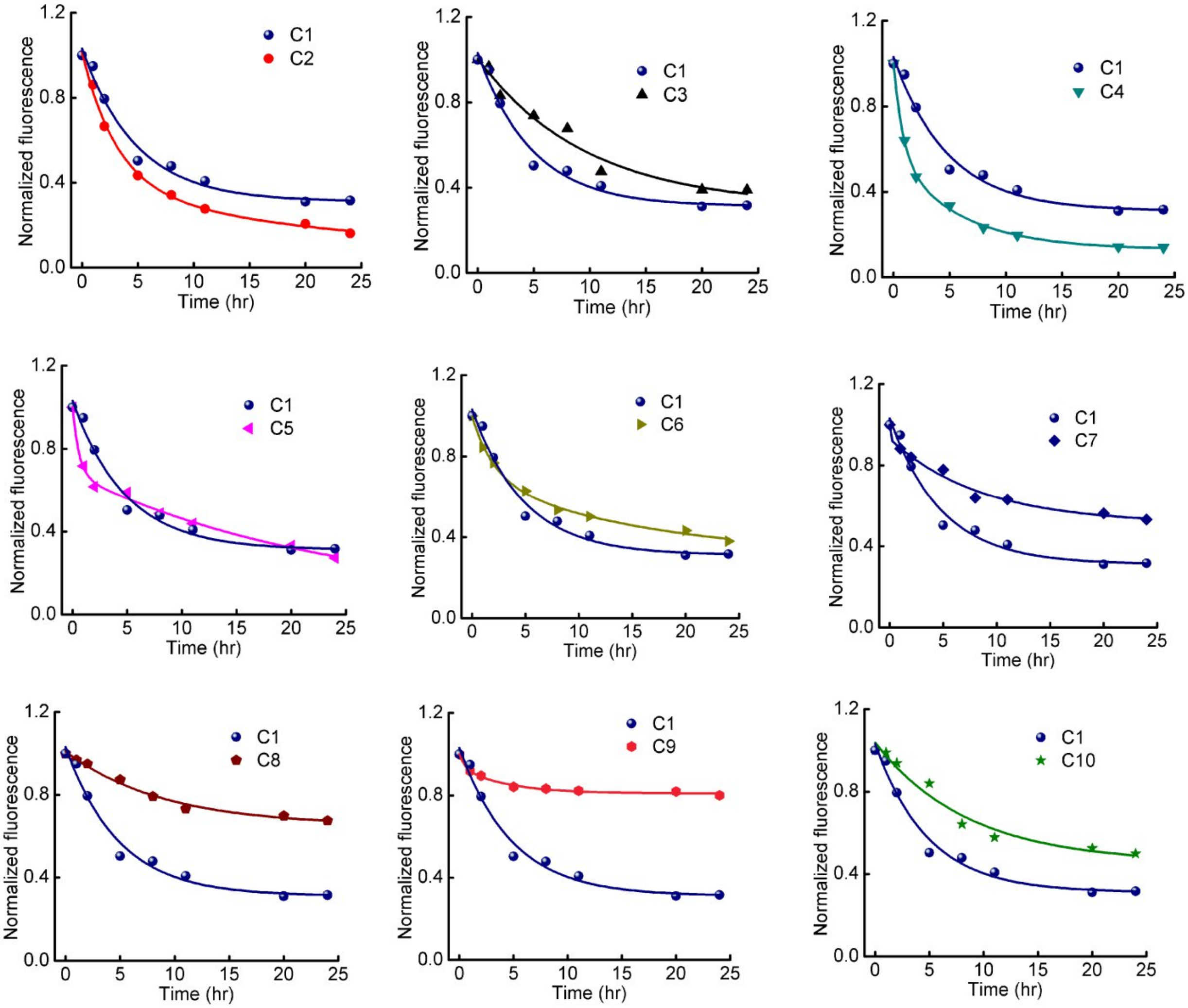
C1
C2
C3
C4
C5
C6
C7
C8
C9
C10
Normalized fluorescence
Time (hr)
0.0
0.4
0.8
1.2
0
5
10
15
20
25

Fig. S3

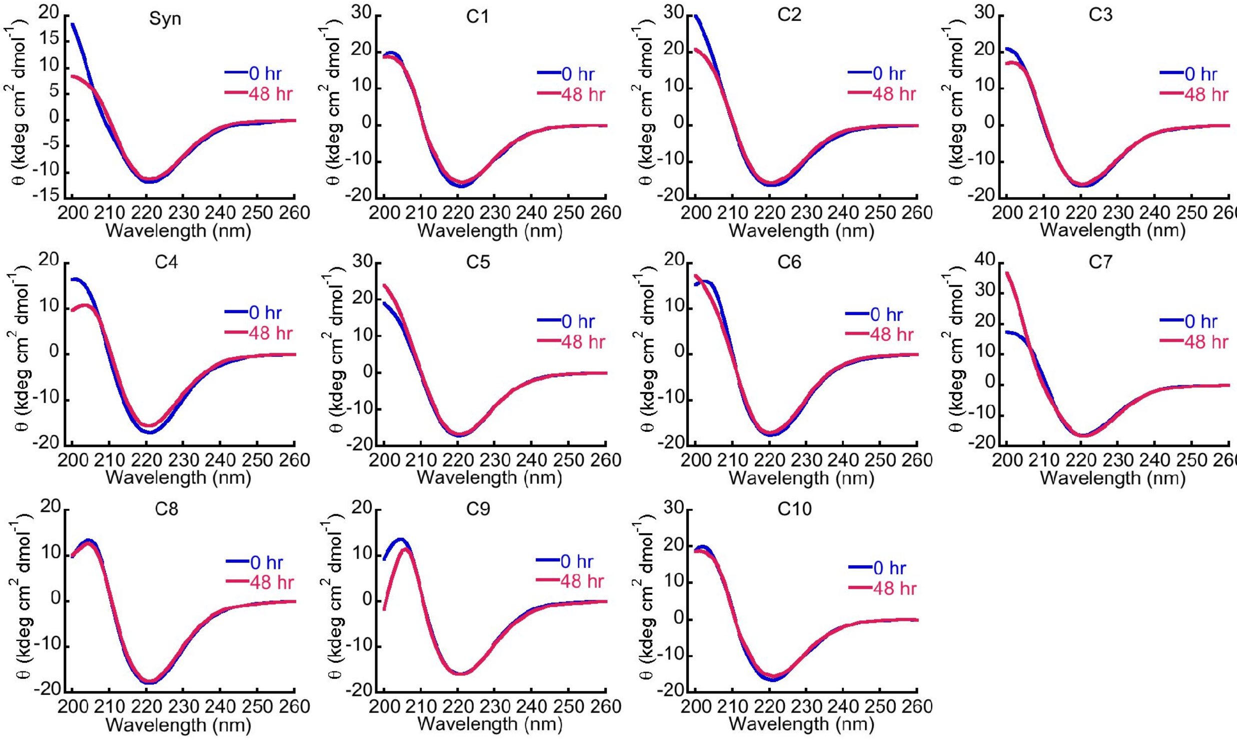
Syn
C1
C2
C3
C4
C5
C6
C7
C8
C9
C10
0 hr
48 hr
θ (kdeg cm2 dmol-1)
Wavelength (nm)
200 210 220 230 240 250 260

Fig. S4

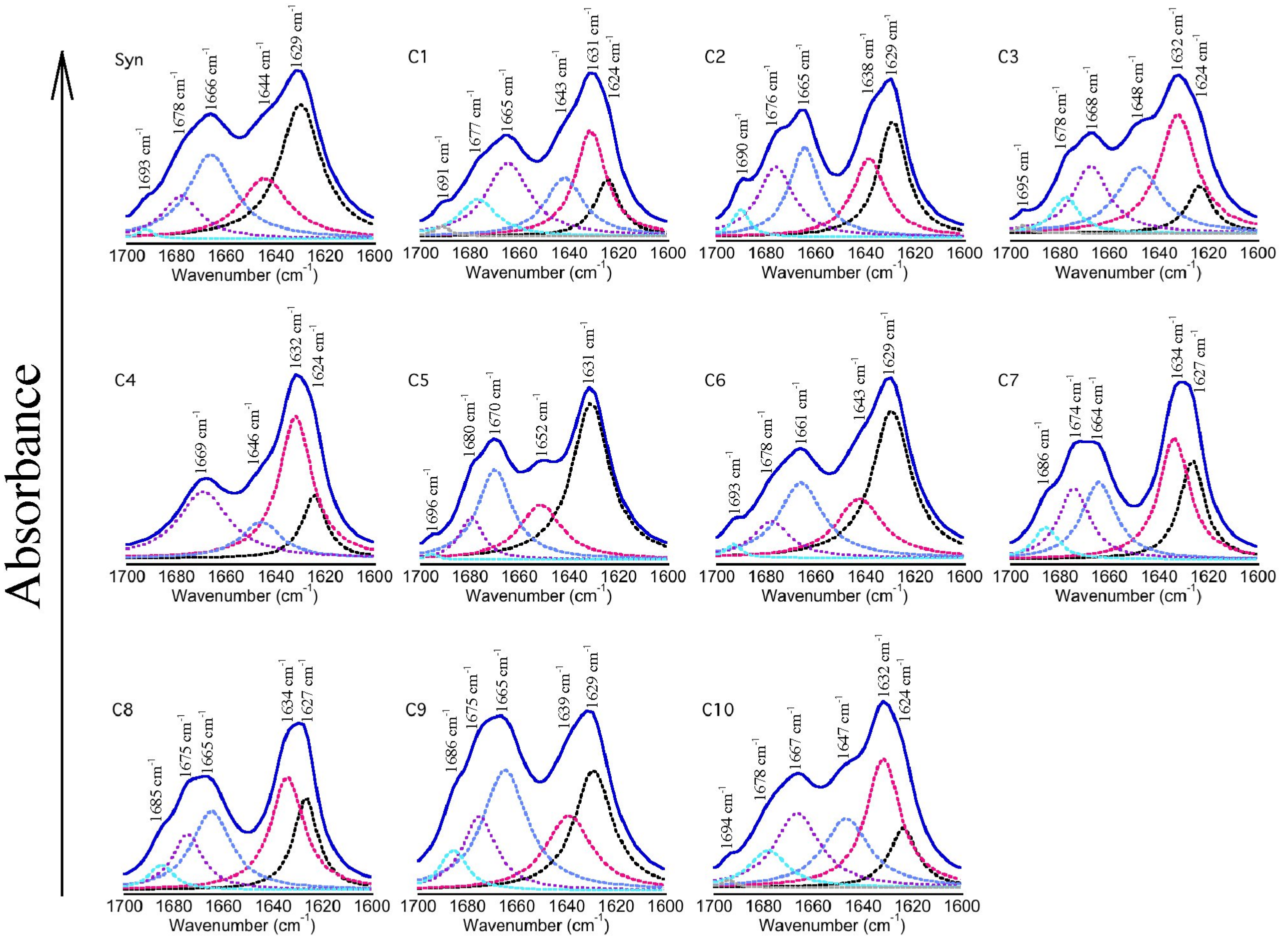

Absorbance
Syn
1693 cm-1
1678 cm-1
1666 cm-1
1644 cm-1
1629 cm-1
C1
1691 cm-1
1677 cm-1
1665 cm-1
1643 cm-1
1631 cm-1
1624 cm-1
C2
1690 cm-1
1676 cm-1
1665 cm-1
1638 cm-1
1629 cm-1
C3
1695 cm-1
1678 cm-1
1668 cm-1
1648 cm-1
1632 cm-1
1624 cm-1
C4
1669 cm-1
1646 cm-1
1632 cm-1
1624 cm-1
C5
1696 cm-1
1680 cm-1
1670 cm-1
1652 cm-1
1631 cm-1
C6
1693 cm-1
1678 cm-1
1661 cm-1
1643 cm-1
1629 cm-1
C7
1686 cm-1
1674 cm-1
1664 cm-1
1634 cm-1
1627 cm-1
C8
1685 cm-1
1675 cm-1
1665 cm-1
1634 cm-1
1627 cm-1
C9
1686 cm-1
1675 cm-1
1665 cm-1
1639 cm-1
1629 cm-1
C10
1694 cm-1
1678 cm-1
1667 cm-1
1647 cm-1
1632 cm-1
1624 cm-1
1700 1680 1660 1640 1620 1600
Wavenumber (cm-1)

Fig. S5

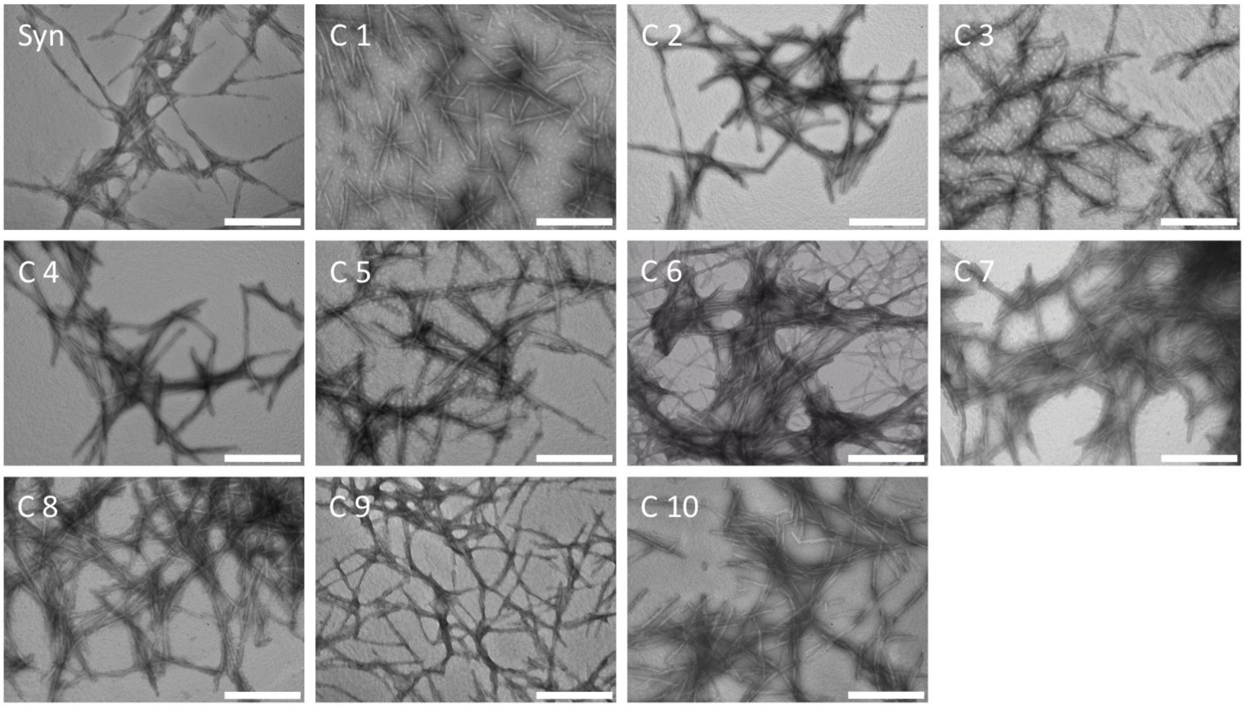

Fig. S6

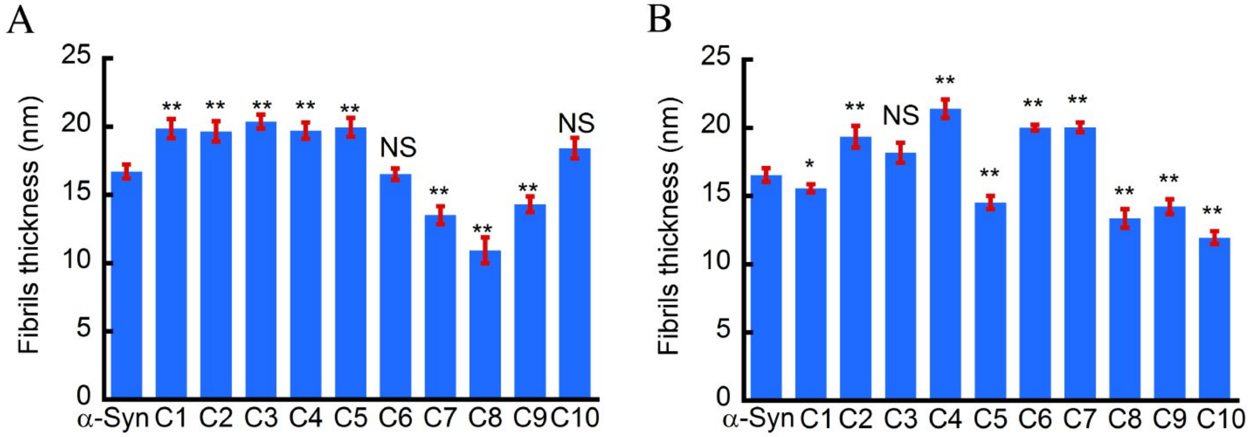
A
B
Fibrils thickness (nm)
25
20
15
10
5
0
α-Syn C1 C2 C3 C4 C5 C6 C7 C8 C9 C10
**
NS
*

Fig. S7

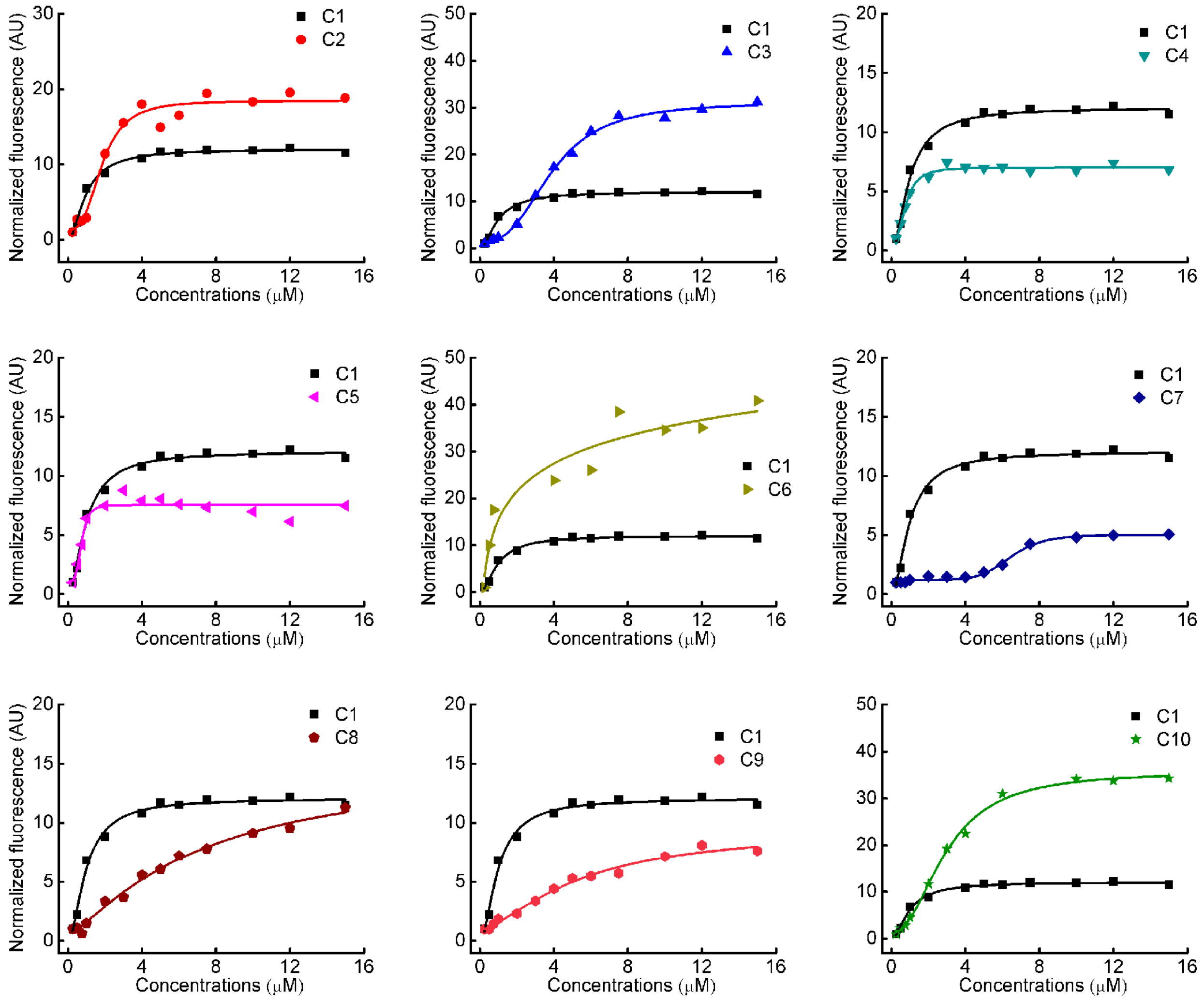

Normalized fluorescence (AU)
Concentrations (μM)
C1
C2
C3
C4
C5
C6
C7
C8
C9
C10

Fig. S8

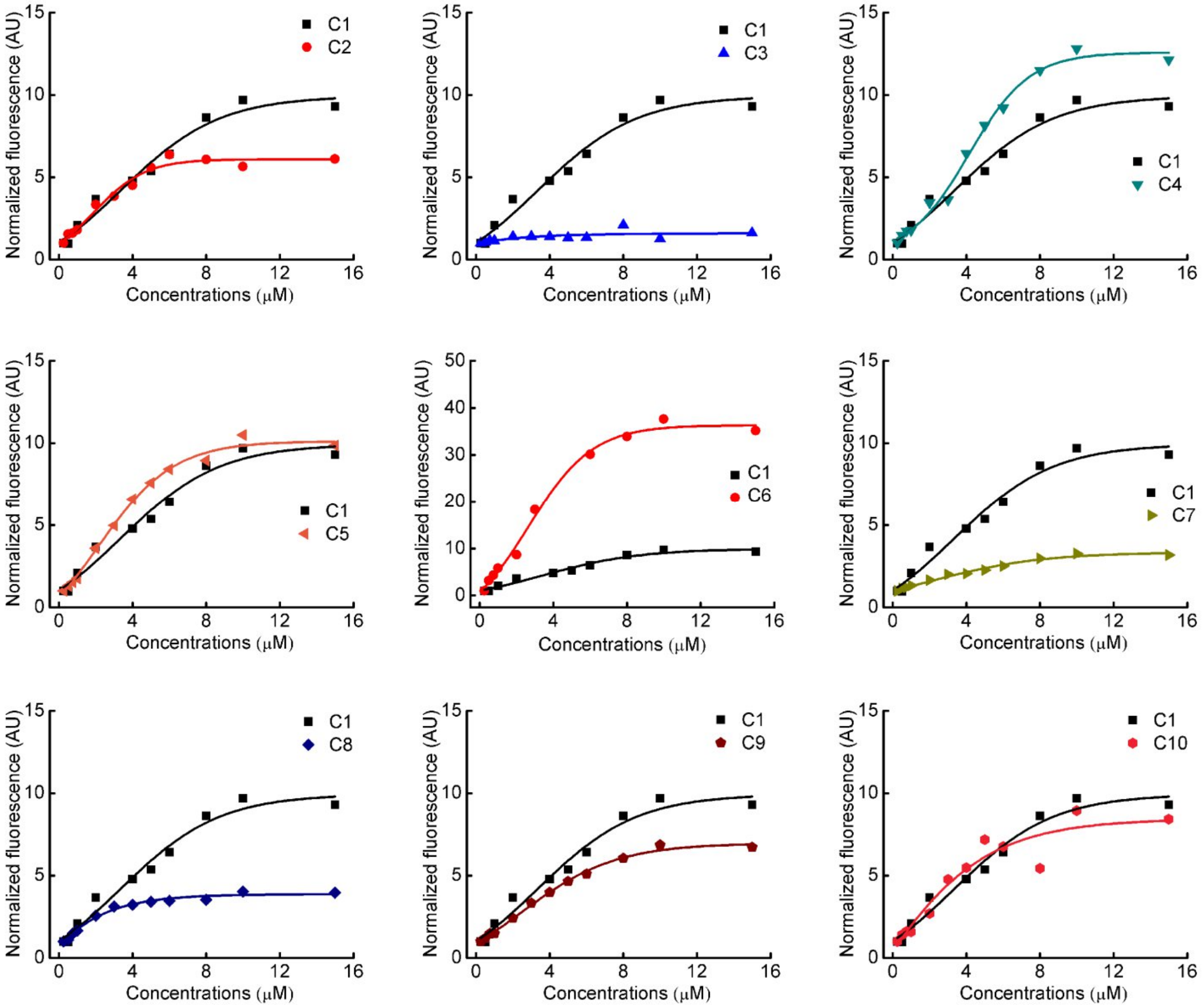

C1
C2
C3
C4
C5
C6
C7
C8
C9
C10
Normalized fluorescence (AU)
Concentrations (μM)

Fig. S9

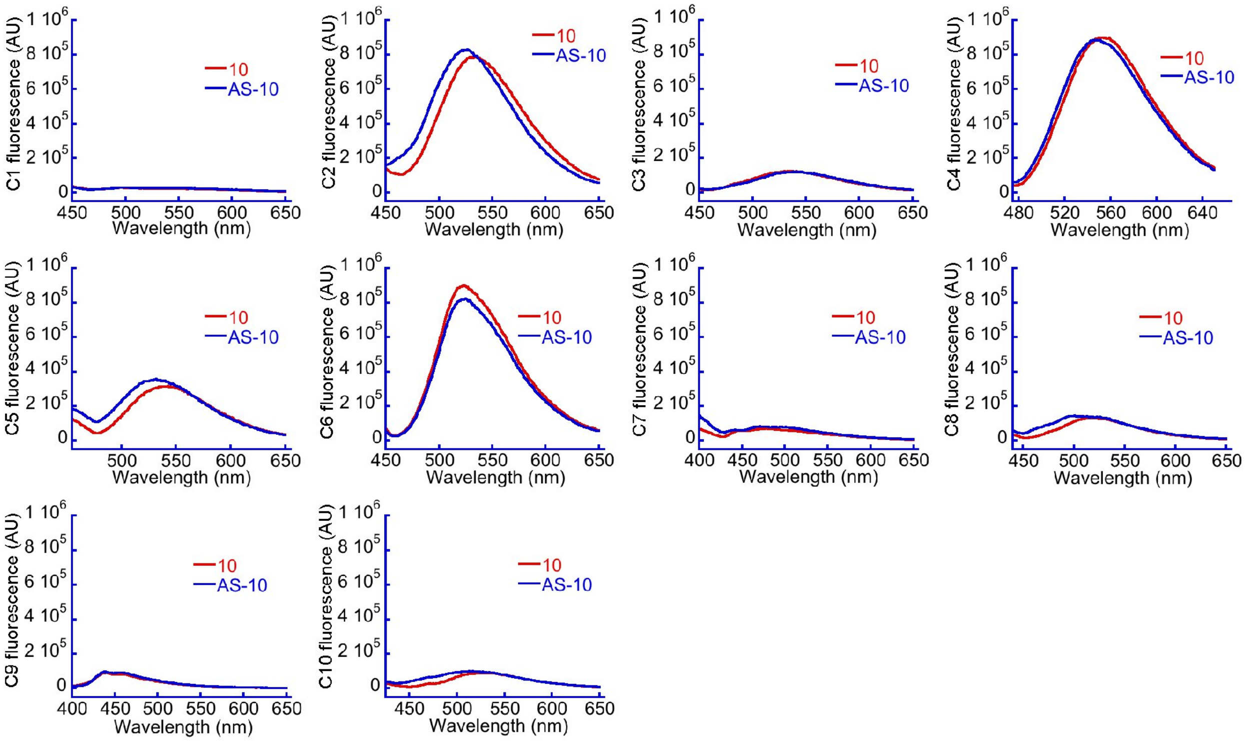

C1 fluorescence (AU)
C2 fluorescence (AU)
C3 fluorescence (AU)
C4 fluorescence (AU)
C5 fluorescence (AU)
C6 fluorescence (AU)
C7 fluorescence (AU)
C8 fluorescence (AU)
C9 fluorescence (AU)
C10 fluorescence (AU)
Wavelength (nm)
10
AS-10

Fig. S10

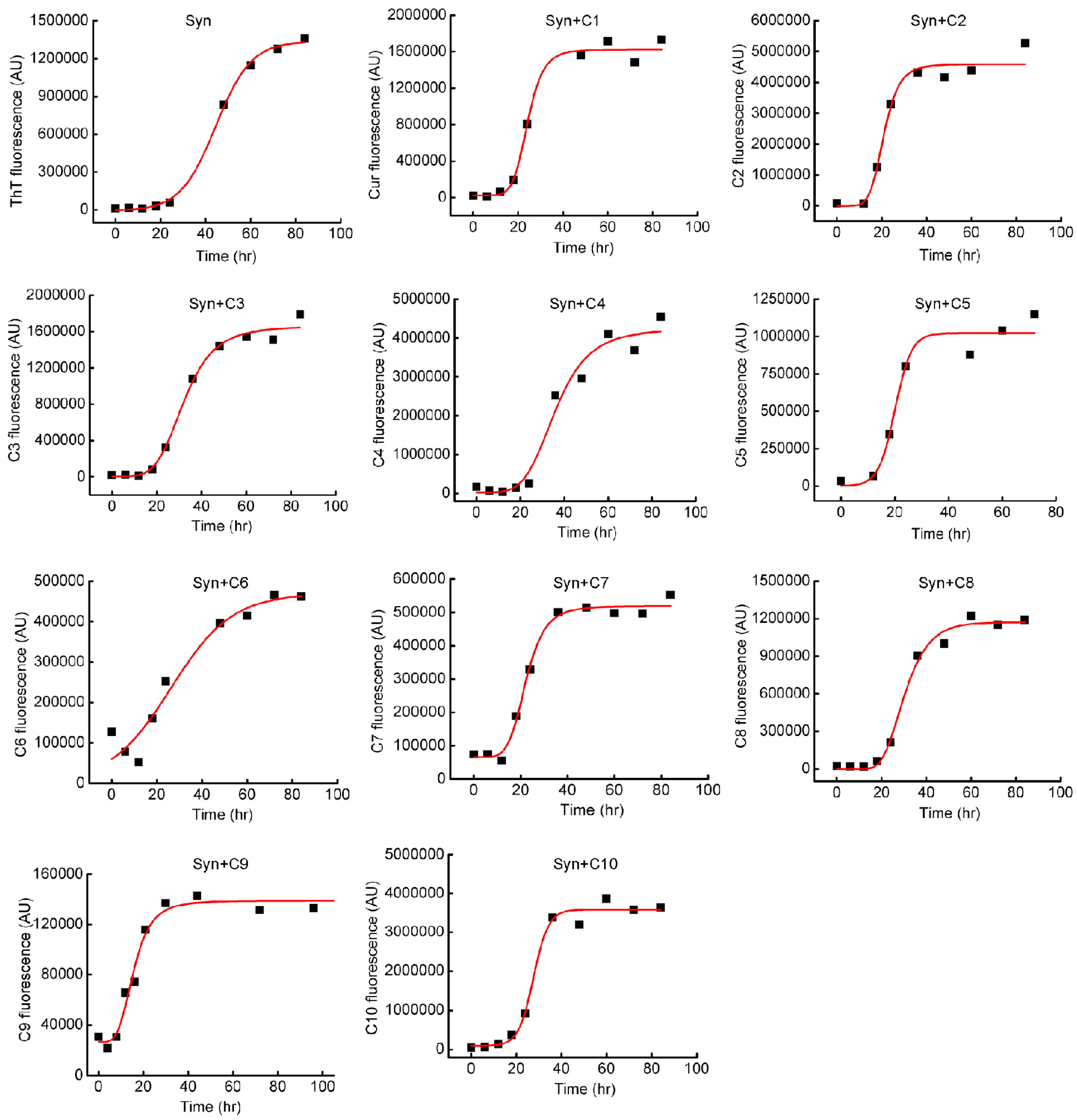
Syn
ThT fluorescence (AU)
Time (hr)
Syn+C1
Cur fluorescence (AU)
Syn+C2
C2 fluorescence (AU)
Syn+C3
C3 fluorescence (AU)
Syn+C4
C4 fluorescence (AU)
Syn+C5
C5 fluorescence (AU)
Syn+C6
C6 fluorescence (AU)
Syn+C7
C7 fluorescence (AU)
Syn+C8
C8 fluorescence (AU)
Syn+C9
C9 fluorescence (AU)
Syn+C10
C10 fluorescence (AU)

Fig. S11

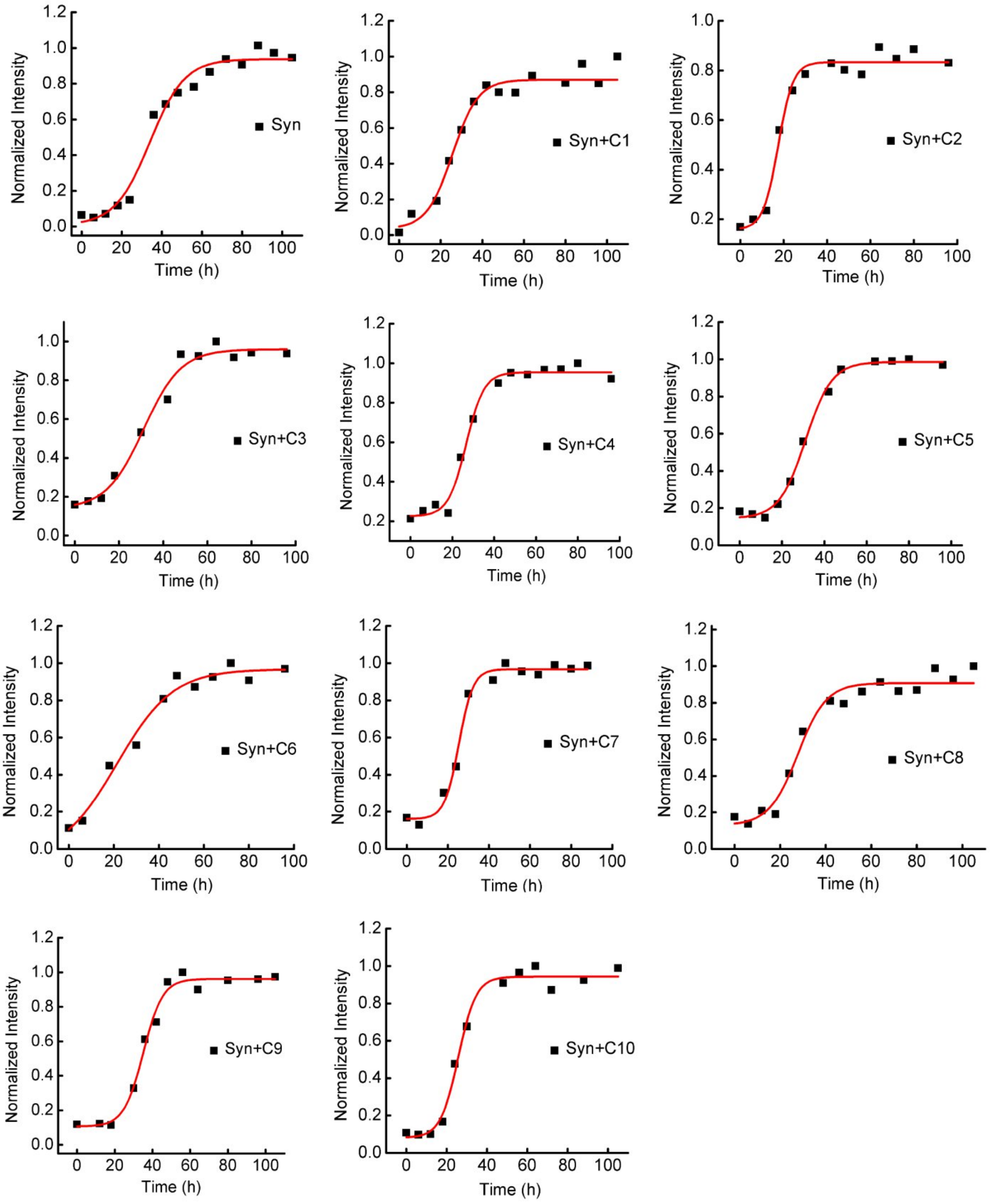

Normalized Intensity
Time (h)
Syn
Syn+C1
Syn+C2
Syn+C3
Syn+C4
Syn+C5
Syn+C6
Syn+C7
Syn+C8
Syn+C9
Syn+C10

Fig. S12

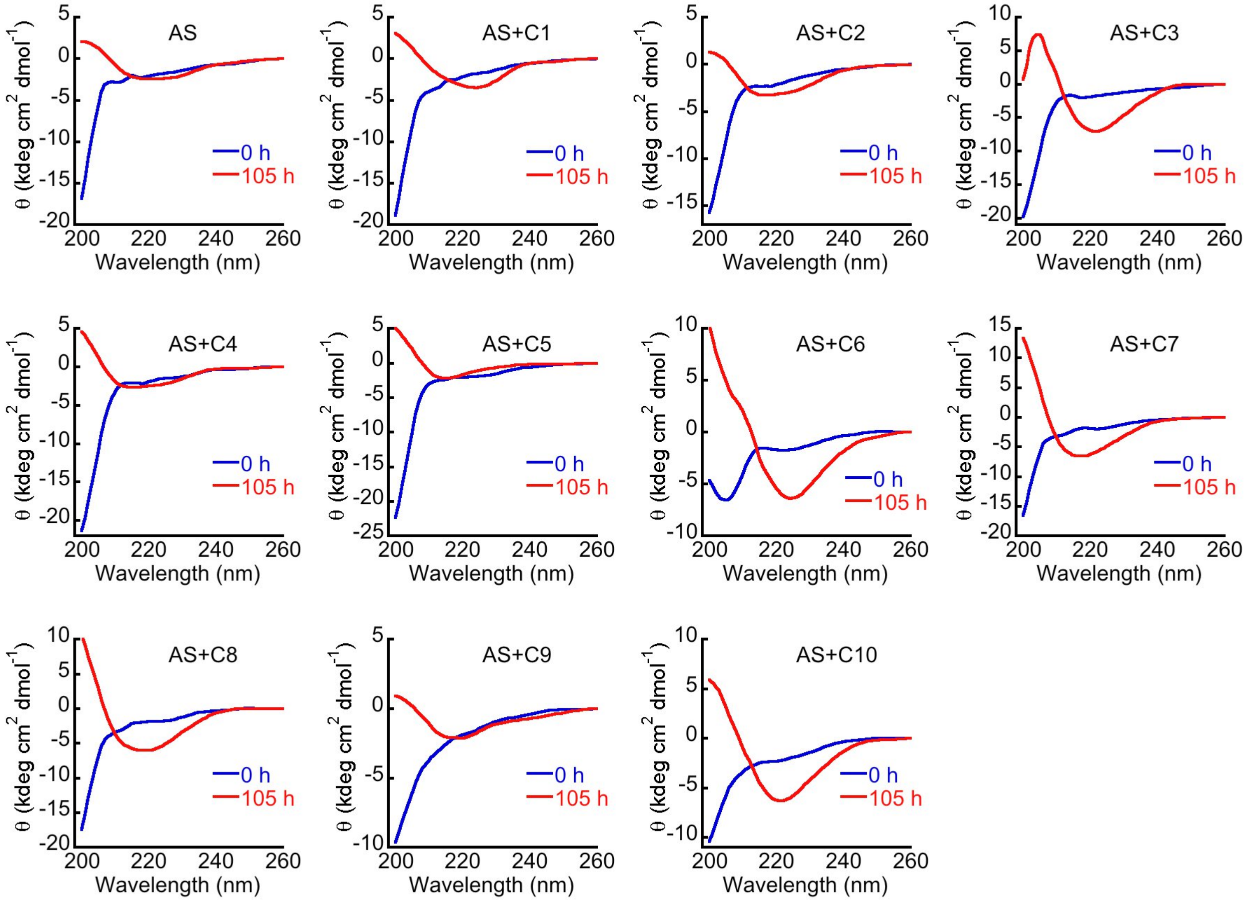
AS
AS+C1
AS+C2
AS+C3
AS+C4
AS+C5
AS+C6
AS+C7
AS+C8
AS+C9
AS+C10
θ (kdeg cm^2 dmol^-1)
Wavelength (nm)
0 h
105 h

Supplementary Tables

Table S1 Percentage of different secondary structures present in the preformed fibrils after 48 h of incubation in absence and presence of curcumin and its analogs. Secondary structural composition was determined by integrating amide-I region (1700-1600 cm^{-1}) of curve fitted FTIR spectra.

Curcumin Analogs	% secondary structure			
	β-sheet	α-helix	Random coil	β-turn
AS	40.6	26.8	21.7	10.7
AS+C1	43.3	26.2	19.9	10.5
AS+C2	55.7	24.7	-	19.5
AS+C3	47.9	19.6	24.4	8
AS+C4	58.9	-	14	27
AS+C5	48	18.5	-	33.3
AS+C6	44.2	24.9	21.2	9.5
AS+C7	59.9	21.9	-	18.1
AS+C8	58.9	26.4	-	14.6
AS+C9	51.2	-	29.9	14.2
AS+C10	45.6	22.6	22.8	8.9

Table S2 Dissociation constants (K_d) values of curcumin and its analogs for α-Syn fibrils and oligomers measured by curcumin and it analogs fluorescence. The dissociation constants (K_d) represent mean±SE from three independent sets of experiments.

Samples	K_d fibrils (μM) (Mean ± SE)	K_d oligomers (μM) (Mean ± SE)
Syn+C1	1.04 ± 0.01	5.83 ± 1.03
Syn+C2	2.61 ± 0.60	2.94 ± 0.90
Syn+C3	7.67 ± 1.97	No Binding
Syn+C4	0.95 ± 0.17	16.46 ± 2.63
Syn+C5	1.01 ± 0.32	6.64 ± 1.45
Syn+C6	0.48 ± 0.07	16.08 ± 3.81
Syn+C7	13.18 ± 2.29	11.95 ± 3.06
Syn+C8	9.64 ± 1.49	1.46 ± 0.20
Syn+C9	2.74 ± 1.97	6.88 ± 0.19
Syn+C10	5.52 ± 1.27	4.15 ± 0.68

Table S3 The lag time for aggregation kinetics of α-Syn in absence and presence of curcumin/analogs obtained from curcumin/analog fluorescence. For α-Syn only lag time was calculated from ThT fluorescence. The values of lag time are represented as mean±SE from three independent sets of experiments.

Samples	Lag Time (h) (Mean ± SE)
Syn	21.5 ± 0.5
Syn+C1	9.5 ± 1.5
Syn+C2	11.0 ± 2.0
Syn+C3	14.0 ± 2.0
Syn+C4	19.5 ± 3.5
Syn+C5	11.0 ± 1.0
Syn+C6	3.5 ± 0.5
Syn+C7	8.5 ± 0.5
Syn+C8	13.0 ± 2.0
Syn+C9	15.0 ± 1.0
Syn+C10	12.5 ± 0.5

Table S4 The lag time for aggregation kinetics of α-Syn in absence and presence of curcumin/analogs obtained from light scattering experiments. The values of lag time are represented as mean±SE from three independent sets of experiments.

Samples	Lag Time (h) (Mean ± SE)
Syn	22.5 ± 1.5
Syn+C1	11.0 ± 3.5
Syn+C2	11.5 ± 2.5
Syn+C3	15.5 ± 2.0
Syn+C4	16.5 ± 3.5
Syn+C5	13.0 ± 1.5
Syn+C6	4.5 ± 0.5
Syn+C7	10.5 ± 1.5
Syn+C8	14.5 ± 2.0
Syn+C9	16.0 ± 2.5
Syn+C10	14.5 ± 2.5
